# Label-free optical observation of disordered-to-ordered transitions in single intrinsically disordered proteins

**DOI:** 10.1038/s44328-026-00098-7

**Published:** 2026-06-02

**Authors:** Saaman Zargarbashi, Cyril Dominguez, Matthew Peters, Arman Yousefi, Sharon Munday, Yanhong Wang, Shreyasi Chatterjee, Andrew J. Hudson, Reuven Gordon, Christopher J. Mellor, Lei Xu, Mohsen Rahmani, Cuifeng Ying

**Affiliations:** 1 Advanced Optics and Photonics Lab, Department of Engineering, School of Science and Technology, Nottingham Trent University, Nottingham, UK; 2 School of Physics and Astronomy, University of Nottingham, Nottingham, UK; 3 Leicester Institute for Structural and Chemical Biology, University of Leicester, Leicester, UK; 4 Division of Molecular and Cell Biology, School of Biological and Biomedical Sciences, University of Leicester, Leicester, UK; 5 Department of Electrical and Computer Engineering, University of Victoria, Victoria, BC Canada; 6 Physical and Theoretical Chemistry Laboratory, Department of Chemistry, University of Oxford, Oxford, UK; 7 Department of Biochemistry, School of Science and Technology, Nottingham Trent University, Nottingham, UK

**Keywords:** Biochemistry, Biophysics, Structural biology

## Abstract

Intrinsically disordered proteins (IDPs) and structured proteins with intrinsically disordered regions (IDRs) lack a definitive tertiary structure and contribute to the onset of diseases such as Alzheimer’s and cancer. To date, experimental observation of single, label-free IDPs/IDRs poses a significant challenge due to their structural heterogeneity, limiting ensemble techniques from fully capturing their properties, whilst single-molecule measurements require site-specific modifications or non-physiological conditions, perturbing their native biophysics. Here, we demonstrate the first experimental observation of unmodified IDP/IDR conformational dynamics at the single-molecule level, achieved by optical trapping and investigation of individual IDPs/IDRs using nanoaperture optical tweezers. Our results reveal that IDPs/IDRs exhibit significantly larger conformational variations compared to globular proteins of similar size. We demonstrate that phosphorylation of native tau-441 by glycogen synthase kinase 3-beta (GSK3β-tau) induces compaction and reduced conformational dynamics. We further observed a disorder-to-order transition during the binding of the N-terminal region of the Src-associated protein in mitosis of 68 kDa (Sam68) to G8.5 RNA. These findings present nanoaperture optical tweezers as a powerful approach to advance our understanding of IDPs/IDRs and further decode their roles in associated diseases.

## Introduction

Intrinsically disordered proteins (IDPs) and intrinsically disordered regions (IDRs) in structured proteins, which constitute approximately 70% of the human proteome^1^, play critical roles in biological processes including neurotransmitter regulation^2^, microtubule regulation^3^, and transcription^4^. Their significant prevalence and roles in the development of various diseases, many of which lack effective treatments and reliable early-stage diagnosis, make IDPs/IDRs important targets for research. Example IDPs include tubulin-associated unit (tau) protein and alpha synuclein, which are implicated in neurodegenerative disorders^5^ such as Alzheimer’s disease^6^ and Parkinson’s disease^7^, respectively. IDP/IDR-related diseases also extend to cancer^8^ such as the IDR Sam68 (Src-associated protein in mitosis of 68 kDa), which has implicated involvement in ovarian, kidney and lung cancers^9^.

IDPs/IDRs are conformationally heterogeneous, dynamically fluctuating between different shapes with variable structure, known as a conformational ensemble^1^. This structural flexibility is often integral to the functions of IDPs, where binding to select targets can induce a particular structure necessary for biological activity. These structures present varying levels of disorder, such as an IDP transitioning to a structured conformation, as seen in disorder-to-order transitions^10,11^, or the IDP/IDR retaining partial or complete disorder upon binding to form fuzzy complexes^12^. Their heterogeneity and absence of a defined folded state render many experimental and computational approaches^13^, which were developed for structured proteins, largely inadequate. Understanding their conformational ensemble and its link to their functions is key to understanding their biophysics.

Ensemble measurements, such as nuclear magnetic resonance^14^, small-angle X-ray scattering^15^, and dynamic light scattering^16^, while very informative, cannot entirely capture the heterogeneity among individual copies of the protein. Structural protein characterisation techniques such as cryogenic electron microscopy (cryo-EM) and X-ray crystallography are suitable for globular proteins, but these techniques yield poor resolution of conformational heterogeneity, leading to low or an absence of electron density in micrographs^17,18^. Additionally, such techniques only capture a snapshot of the protein’s motion on its conformational landscape, losing dynamic information, which is integral to IDPs/IDRs. Notably, cryo-EM is progressing towards overcoming some of these limitations, such as the advent of single particle cryo-EM^19^ and the use of artificial intelligence, to enable mapping of flexible areas^20^.

Single-molecule techniques such as single-molecule fluorescence resonance energy transfer (smFRET) and single-molecule force spectroscopy (smFS) can provide excellent information on globular proteins, including free-energy landscapes^21^, tracking intramolecular displacement^22^, and conformational dynamics^23^. These label-based single-molecule approaches have even provided insight into labelled IDPs/IDRs over a decade ago^24,25^. However, the requirement of labelling the protein, such as with an extrinsic fluorophore for smFRET, or tethering the protein to a surface for smFS, can perturb the structure and dynamics of the protein^26,27^, particularly for IDPs/IDRs where many are observed to undergo disorder-to-order transitions upon binding^10,11,28^. Whilst several labelling approaches have been used to investigate IDPs/IDRs, there is a lack of label-free approaches to avoid these limitations. Currently, no established protein characterisation technique can capture the conformational dynamics of label-free IDPs/IDRs at the single-molecule level.

Nanoaperture optical tweezers (NOTs) utilise localised surface plasmon resonance to trap a single protein molecule and observe its conformational changes in physiological conditions without any chemical modifications^29,30^. In recent years, NOTs have provided information on single, label-free proteins, including protein-binding interactions^31^, conformational transitions^32^, disassembly kinetics^33^ and energy landscapes^34^. Here, we utilise NOTs to monitor the conformational dynamics of label-free IDPs/IDRs in solution, elucidate their free-energy landscapes, and observe a disorder-to-order transition of an IDR upon binding to RNA. We focus on three different IDPs/IDRs associated with disease progression: native tau-441, tau-441 phosphorylated by glycogen synthase kinase 3-beta (GSK3β-tau) and the N-terminal region of Sam68. This work reveals differences in structural flexibility between single IDPs and globular proteins of similar size, provides experimental evidence of structural changes due to phosphorylation of IDPs, and the trajectory of a single IDR transitioning from disordered to ordered structures upon binding of a nucleic acid-binding partner. All the above insights into the conformational dynamics of label-free IDPs/IDRs were previously inaccessible to any single-molecule approach.

## Results and Discussion

### Transmission traces distinguish ordered and disordered structures

**Fig. 1:**
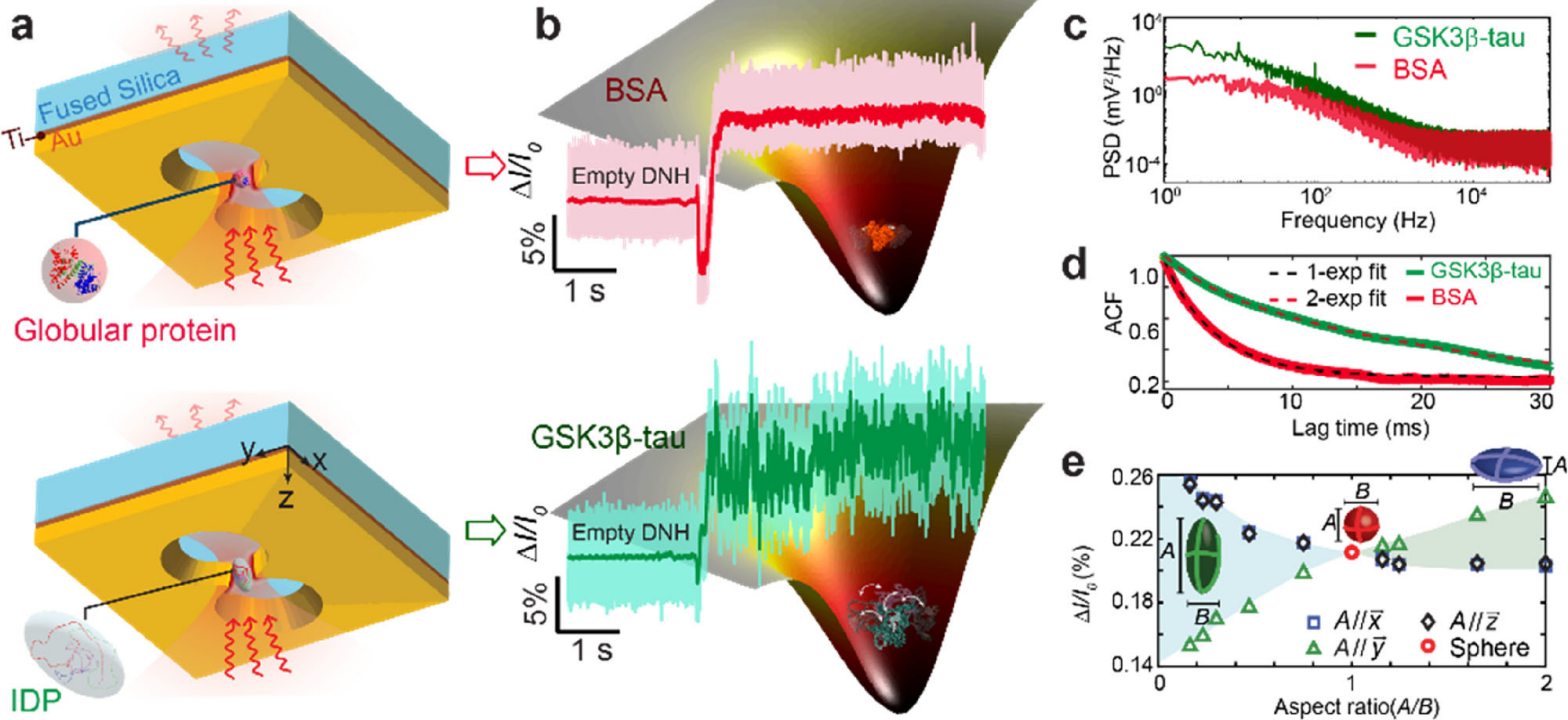
Single-molecule protein trapping using nanoaperture optical tweezers. **a** Schematic of label-free trapping of either a globular protein (top) or an IDP (bottom) within a DNH structure. The DNH was fabricated in a 100-nm gold film and passivated with PEG-thiol (see methods section for detailed parameters). SEM images of representative DNH geometries are shown in Fig. S5. IDP and globular protein structures were generated using AlphaFold 3^73^ and edited with ChimeraX^74^. **b** Representative trapping trajectories for a globular protein (BSA) and an IDP (GSK3β-tau), shown as raw data (sampling rate: 1 MHz) and after low-pass filtering (1 kHz). The schematics illustrate the trapping potential with a protein confined within the well. The globular protein is restricted to lateral and rotational motions within the trap, whereas the IDP undergoes additional conformational fluctuations superimposed on these movements. **c** Power spectral density (PSD) plots of trapping traces for BSA and GSK3β-tau. **d** Autocorrelation functions (ACF) of trapped BSA and GSK3β-tau traces, with exponential decay fits shown as dashed curves. BSA traces follow a single-exponential decay with a time constant of 3.67 ± 0.10 ms, corresponding to a trapping stiffness of ~0.15 fN/nm, whereas GSK3β-tau traces require a double-exponential fit with time constants of 3.56 ± 1.0 ms and 30.24 ± 3.80 ms. **e** Dependence of normalised current change (Δ*I/I*_*0*_) on the orientation and aspect ratios (*A*/*B*) of ellipsoids with axes (*A*, *B*, *B*). Further details for the simulation parameters are provided in the supplementary information (SI-3 and Table S2).

Trapping of either an IDP/IDR or a globular protein is achieved using a gold double-nanohole (DNH) structure (Fig. 1a; full setup in Fig. S1). When a molecule enters the trapping region, the disparity in the refractive index between the trapped molecule and the surrounding media affects the light scattered by the nanoaperture. This scattering directly correlates to the polarisability of the trapped protein, which is determined by its volume, conformation, and dielectric constant^35,36^. The forward-scattered light is collected by an objective (NA = 0.1) and is then recorded as transmission intensity (*I*) by an avalanche photodiode (APD)^37^ (Fig. S1). We analyse protein dynamics using the normalised transmission intensity change, Δ*I/I*_*0*_, where *I*_*0*_ is the baseline intensity of an unoccupied DNH, and Δ*I* = *I* − *I*_*0*_ represents the change in transmission intensity from this baseline (Fig. 1b). In this work, we trap all proteins with the laser power that produces a local temperature of ~37 °C (i.e., 20 mW, see Fig. S2) at the trapping site, as it falls within the range for physiological human body temperature^38^. All globular proteins used in this work are functionally stable at this temperature^39–43^, and IDPs are known for their high thermal stability, due to their low sequence complexity and lack of structure^44–47^.

Comparing representative trapping trajectories for an IDP and a globular protein reveals distinct dynamic behaviours (Fig. 1b). The initial increase in Δ*I/I*_*0*_ corresponds to the protein entering the DNH trap, with the signal magnitude linearly scaling with protein size (Fig. S3d)^33,48^. The observed dip before the trapping event is believed to arise from changes in transmission based on the distance of the trapped particle from the DNH surface before stabilising within the potential well (Fig. S3e)^34^. Due to the lateral and rotational movements of the trapped proteins within the potential well, we observed a significant increase in signal fluctuation (Fig. 1b). For the globular protein BSA, these motions account for most of the signal variations, whereas the IDP GSK3β-tau displays substantially larger fluctuations, reflecting additional conformational dynamics superimposed on its translational and rotational movements. This trend is observed across multiple trapping traces involving various IDPs/IDRs and globular proteins (Figs. S4a, b) rather than being specific to tau and BSA. Figure S4c confirms that IDPs/IDRs demonstrate higher normalised root-mean-square (NRMS) values, and therefore are more dynamic, than globular proteins of similar molecular weight. These enhanced fluctuations, reflecting the highly dynamic structural transitions of IDPs/IDRs, occur predominantly below 1 kHz, as shown in the power spectral density (PSD) plot (Fig. 1c). Using a globular protein as a control allows us to decouple intrinsic protein dynamics from trapping dynamics arising from translational diffusion within the optical potential well. Figure 1d reveals that the autocorrelation function of BSA follows a single exponential decay, yielding a time constant 3.67 ± 0.10 ms. This value leads to an estimated trapping stiffness of 0.15 fN/nm, following the methodology outlined in SI-3 of previous work^31^. However, GSK3β-tau follows a double exponential decay yielding a fast component similar to BSA (~3.6 ms) associated with trap relaxation, and a second, slower exponential decay component with a time constant of ~30 ms, corresponding to its intrinsic conformational transitions. Due to the large conformational dynamics of IDPs, the trapping stiffness cannot currently be resolved as it can for globular proteins, which we use here as a control to verify the trap.

Two factors may contribute to the large optical signal variations observed in IDPs. First, the loose, extended conformation of IDPs exposes a greater surface area to the solvent, resulting in a larger hydration shell with higher water density than that of globular proteins^49,50^. This hydration effect increases the local refractive index around the protein in its elongated states, leading to greater transmission changes. Second, elongated particles of equivalent volume produce orientation-dependent Δ*I/I*_*0*_ signals, as demonstrated by our simulations (Fig. 1e, see SI-3 for details of FDTD simulation). IDPs/IDRs continuously switch between different extended conformations on microsecond-to-second timescales^51^, producing intrinsic Δ*I/I*_*0*_ fluctuations that reflect their conformational sampling and orientations. We hypothesise that elongated globular proteins may preferentially adopt a trapping orientation that maximises the local electric field through the self-induced back action (SIBA) trapping mechanism, and consequently, results in a higher average Δ*I/I*_*0*_ value than their spherical counterparts, as previously reported^31,34,52^. As detailed in the supplementary information (SI-3 and SI-5), Δ*I/I*_*0*_ depends critically on the refractive index, shape, and orientation of the protein within the DNH gap. These dependencies enable Δ*I*/*I*_*0*_ to serve as an indicator for the global compactness of the trapped protein.

### Phosphorylation-induced order of tau by GSK3β

**Fig. 2:**
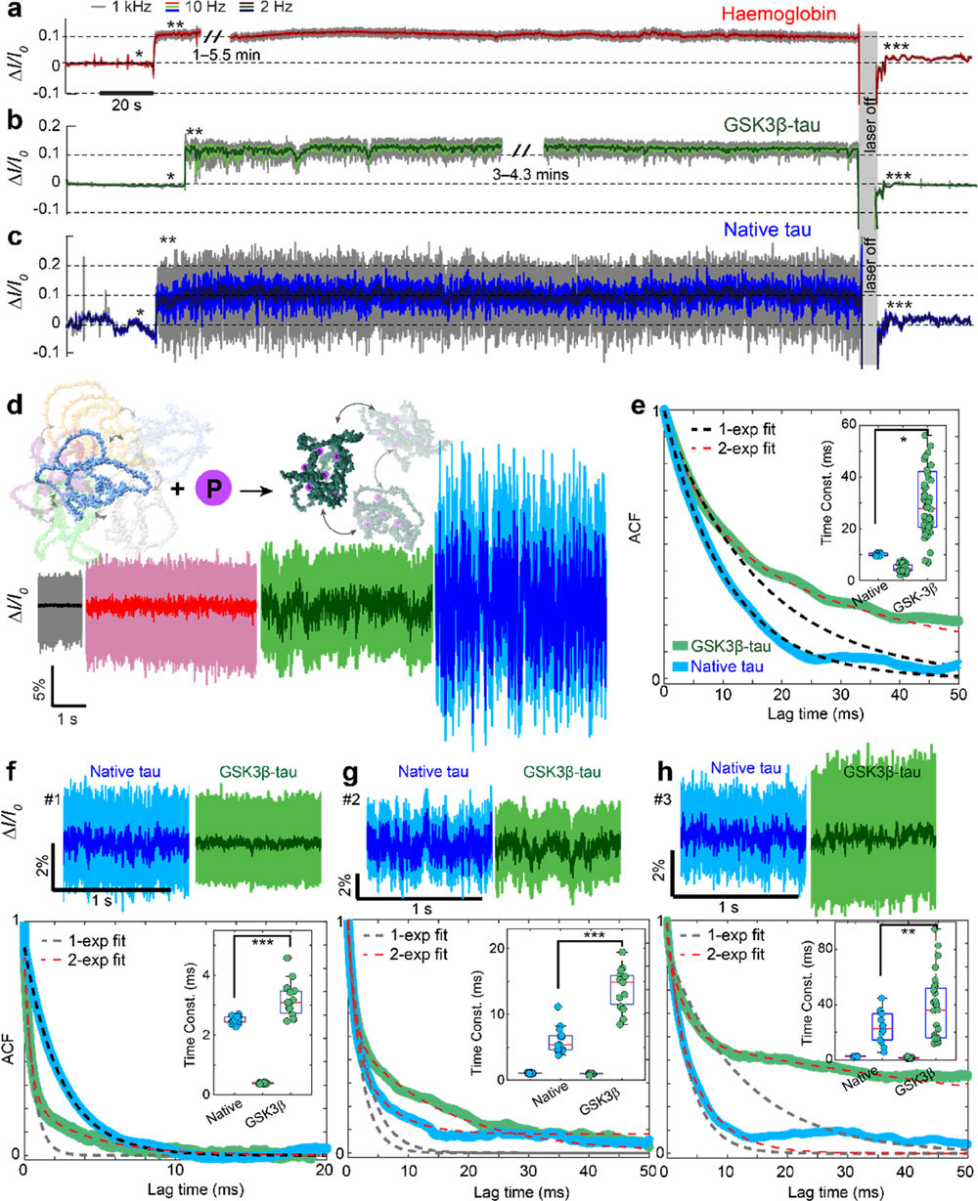
Effect of GSK3β phosphorylation on tau-441. Optical transmission traces over 6 min through a double nanohole (DNH) for trapped proteins: **a** haemoglobin; **b** GSK3β-tau; and **c** native tau-441. Transmission intensities were sampled at 1 MHz and digitally filtered at 1 kHz (grey), 10 Hz (light coloured) and 2 Hz (dark coloured). Raw traces are shown in Fig. S9. Asterisks indicate the following events: baseline before trapping (*), protein trapped (**), and protein released (***). **d** Zoomed segments taken from (**a–c**), comparing native tau-441 (blue), GSK3β-tau (green), haemoglobin (red), and baseline (black). Data are shown in raw (1 MHz) and filtered (1 kHz). Inset: Schematic illustration showing that phosphorylation reduces the degree of disorder in the tau protein. The depicted protein structures are shown only for illustrative purposes. IDP structures were generated using Alphafold 3^73^ and edited with ChimeraX^74^. **e** Autocorrelation functions (ACF) of trapping traces for native tau (blue) and GSK3β-tau (green) proteins, along with their exponential decay fits, with single-exponential fits shown as black dashed curves and double-exponential fits as red dashed curves. Inset: Time constants from exponential fits to the autocorrelation curves. **f–h** Top: one-second transmission traces from three independent experiments comparing native tau (blue) and GSK3β-tau (green), shown as both raw (1 MHz,) and filtered (1 kHz). Bottom: Corresponding ACF plots for each experiment, along with their exponential decay fitting (dashed curves) and time constants. Dataset #1 was obtained using a third-party fabricated DNH with a reduced gap size (see red box in Fig. S5 for SEM images).

Figures 2a–c compare the transmission-time traces corresponding to the trapping of the IDP native tau-441 and its phosphorylated variant GSK3β-tau, with those of the globular protein haemoglobin. Trapping these proteins using DNHs with similar dimensions resulted in comparable Δ*I*/*I*_*0*_ of approximately 0.1 (Fig. 2a–c), due to their similar molecular weight (native tau-441, 45.9 kDa, GSK3β-tau, ~46–48 kDa, and haemoglobin, 64.5 kDa). The extended conformations of IDPs result in higher polarisability than globular proteins of similar molecular weight, which explains why native tau-441 and GSK3β-tau exhibit Δ*I/I*_*0*_ values comparable to haemoglobin despite their lower molecular weight. The trace segments in Fig. 2d reveal substantially different fluctuations in Δ*I/I*_*0*_ between the three proteins. Native tau-441 and GSK3β-tau resulted in larger fluctuations compared to haemoglobin, consistent with the higher flexibility of IDPs relative to globular proteins. When the laser is turned off for several seconds and then turned back on, the transmission intensity returns to the baseline level (Fig. 2a–c), indicating that the protein molecule diffused away from the trap without surface adsorption. These results demonstrate the effectiveness of the PEG-thiol coating in minimising nonspecific protein adsorption, a major challenge when studying IDPs/IDRs, particularly under conditions where the trapping force retains them close to the gold surface for extended periods. Occasionally, however, the protein did not diffuse away after the laser was turned off, as shown in Fig. S6. Trapping data after these events occurred were excluded from subsequent analysis and discussion in this work. Additionally, we compare and discuss the sticking frequency of the proteins used in this work, where the IDPs/IDRs demonstrated a higher propensity to stick compared to the globular proteins tested (Table S3 and SI-7).

The results shown in Fig. 2a–d suggest that GSK3β phosphorylation of native tau-441 induces increased order and compaction. Two mechanisms likely underlie these changes: electrostatic interactions and secondary structure formation. First, native tau-441 has a theoretical isoelectric point of ~8.24, carrying a net positive charge at pH 7.2. Phosphorylation introduces negatively charged phosphate groups, subsequently reducing the net charge and promoting compaction, consistent with previous reports^53,54^. Second, phosphorylation may shift local secondary structure. GSK3β phosphorylation has been shown to increase α-helix propensity at the expense of polyproline type II (PPII) helices within the proline-rich domain of tau^55^ (Fig. S7). As α-helices are shorter than PPII helices (5.4 Å/turn vs. 9.3 Å/turn)^56^, this structural transition would also induce compaction in native tau-441. Supplementary Section SI-8 provides an in-depth discussion of potential phosphorylation effects.

Analysis of the autocorrelation functions for native tau-441 and GSK3β-tau (Fig. 2e) revealed an additional secondary decay (~26 ms) in the phosphorylated variant. In contrast, native tau-441 exhibited only a single exponential component (~10 ms), likely arising from the similar timescales of confined trapping dynamics and conformational fluctuations. To confirm that GSK3β phosphorylation shifts native tau-441 towards more compact and ordered conformations, we measured data from three additional trapping experiments recorded in DNHs with the same/similar geometries, shown in Fig. 2f–h (five second variants of these traces are shown in Fig. S8a–c). GSK3β-tau consistently exhibited reduced dynamic behaviour compared to native tau-441, demonstrated by less variation in Δ*I/I*_*0*_. While the ACF for native tau-441 could sometimes be fit with a double-exponential decay, suggesting separable conformational and trapping dynamics, GSK3β-tau consistently exhibited a slower time constant. Compared to native tau-441, the PSD of GSK3β-tau (Fig. S8d) displays lower power fluctuations in the 1 Hz–1 kHz range, indicating its increased order and restricted large-scale dynamics occurring on the second–millisecond timescale. These results suggest that GSK3β phosphorylation introduces structural order into tau. Rather than fluctuating continuously among disordered states on millisecond timescales, the phosphorylated variant samples more confined conformations that fluctuate over tens of milliseconds. We note that the number and location of phosphorylated residues in GSK3β-tau may vary between molecules. Consequently, this may contribute to the heterogeneity observed in the trapping trace patterns among individual GSK3β-tau molecules.

**Fig. 3:**
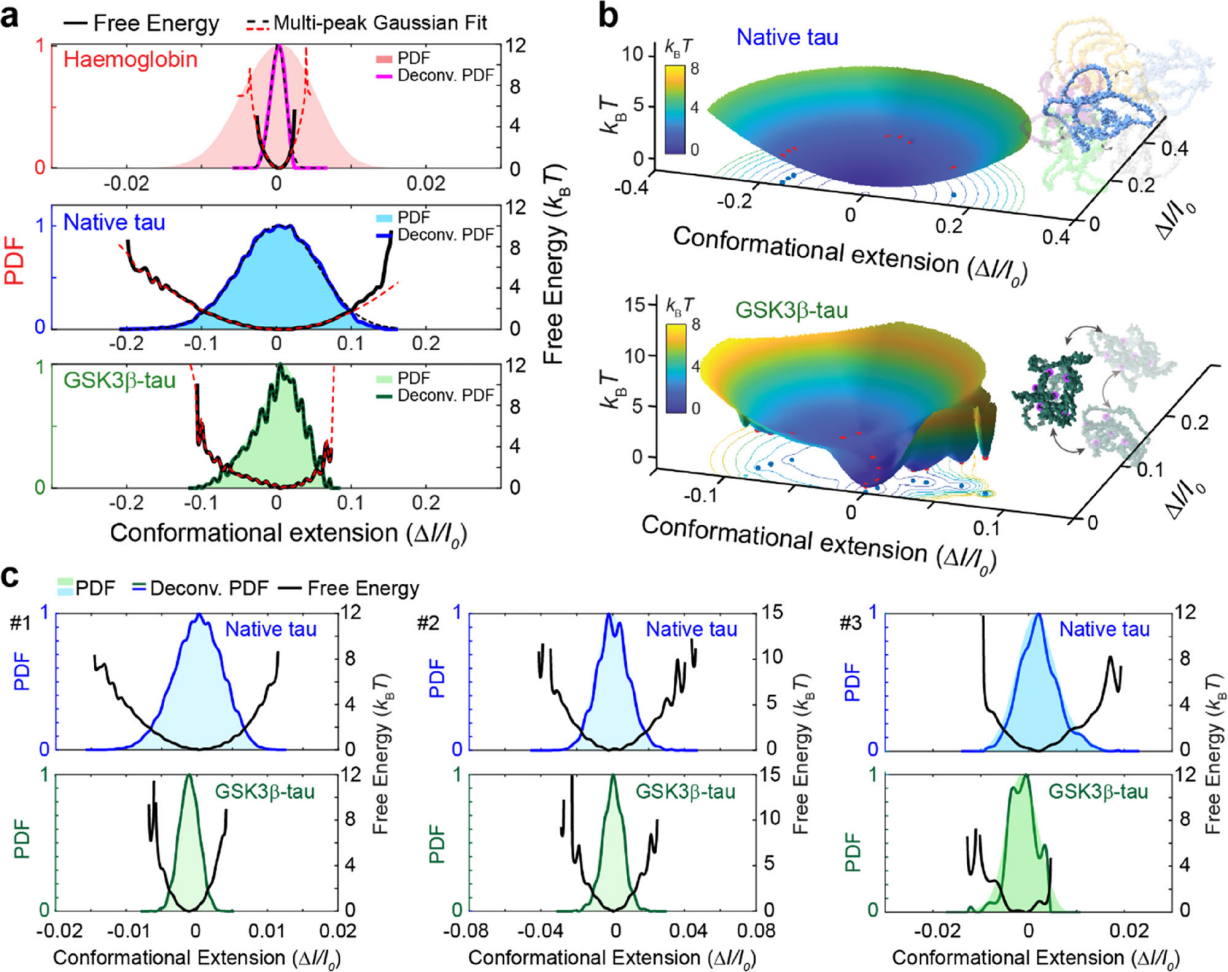
Revealing protein dynamics and energy landscapes using nanoaperture optical tweezers. **a** Probability density functions (PDFs) of Δ*I/I*_*0*_ over 20 s of 10 kHz digital filtered data for each protein (colour shaded regions), with deconvoluted PDFs fitted using multi-peak Gaussian models (black dashed curves). PDFs were deliberately overfitted to maximise accuracy in reconstructing the free-energy landscapes (Fig. S10a, b). 1D energy landscapes were derived from the deconvoluted PDFs for haemoglobin (red line), GSK3β-tau (green line), and native tau-441 (blue line). **b** 2D energy landscapes over 20 s for GSK3β-tau and native tau-441, constructed from the deconvoluted PDFs in (**a**). Distance from the origin (0, 0, 0) reflects conformational extension (Δ*I/I*_*0*_ magnitude, *x*- and *y-*axis) and thermodynamic stability (*k*_B_*T, z-*axis). See Supplementary Section SI-11 for details on PDF deconvolution and the calculation of energy landscapes. The protein structures were added for illustration purposes. IDP structures were generated using Alphafold 3^73^ and edited with ChimeraX^74^. **c** PDFs of Δ*I/I*_*0*_ over 20 s of 10 kHz digital filtered data for three additional datasets (colour shaded regions), along with deconvoluted PDFs (coloured curves) and derived free energy landscapes.

Probability density functions (PDFs) of the transmitted intensity reveal how frequently a protein samples different conformations, providing valuable insight into its accessible structural states. Deconvolution of the PDFs using a point spread function removes the translational and rotational motion of proteins and allows extraction of the PDFs associated with the protein’s true conformational changes. Figure 3a shows that the deconvoluted PDF for haemoglobin reveals a single, sharp peak, consistent with it existing predominantly in a single conformational state, characteristic of a globular protein. In contrast, the deconvoluted PDFs of both GSK3β-tau and native tau-441 exhibit broader Δ*I/I*_*0*_ distributions, indicating greater conformational variability and dynamic behaviour than haemoglobin. Native tau-441 displays the broadest distribution, suggesting it samples a wider conformational landscape and possesses greater structural flexibility than GSK3β-tau.

Converting the PDF of single-molecule folding trajectories into free-energy landscapes is well established in techniques such as FRET^57–59^ and smFS^60^. These landscapes provide valuable insights into protein folding, including the number of distinct conformational states, their relative free-energy differences, and how these are altered by binding interactions^61^. Recently, NOTs have been used to resolve the energy landscape of a single unmodified protein^34^. For label-free monomeric IDPs/IDRs, experimental derivation of energy landscapes is particularly important for understanding their conformational dynamics, molecular interactions, and biological functions^61^. Until now, this information has been accessible only through computational modelling^62–64^, due to the challenges of experimentally measuring label-free IDPs/IDRs at the single-molecule level.

Here, we present the first experimentally derived measurement of the free-energy landscape of label-free IDPs at the single-molecule level. 1D energy landscapes were calculated by taking the negative logarithm of the deconvoluted PDFs, as described by Eq. S3, and are shown in Fig. 3a, c (black curves). Relative free-energy values are expressed in units of *k*_B_*T*, where *k*_B_ is Boltzmann’s constant and *T* is temperature, representing the energy available to the protein. As expected, haemoglobin exhibits a sharp funnel-like landscape characteristic of globular proteins, whereas native tau-441 and GSK3β-tau display multiple shallow minima with lower energy barriers between them, consistent with a broad ensemble of conformational states typical of IDPs^65^. Across all experiments, the deconvoluted PDFs and energy landscapes of GSK3β-tau revealed more compact conformational ensembles, consistent with their smaller Δ*I*/*I*_0_ fluctuations.

To better visualise the complex conformational dynamics of these IDPs, we converted the 1D energy landscapes into 2D plots. First, we applied multi-peak Gaussian fits to the deconvoluted PDFs to identify predominant conformational states (black dashed curves in Fig. 3a, with individual peaks presented in Fig. S10a). These fitted PDFs were then used to reconstruct the corresponding energy landscapes (black dashed curves, Fig. 3a), which were further converted to 2D plots (Fig. 3b) by mapping the peak positions onto a polar coordinate system (Fig. S11). In these plots, angular coordinates reflect the relative spatial distribution of conformational states, while the peak amplitudes correspond to the negative logarithm of the fitted PDF, such that a higher probability indicates a lower free energy. Haemoglobin, as expected for a globular protein, displays a single, funnel-shaped energy landscape consistent with a single, stable folded conformation (Fig. S12). For native tau-441, the 2D energy landscapes reveal multiple shallow troughs with low energy barriers, consistent with the conformational heterogeneity expected in IDPs. In contrast, alongside shallow troughs similar to those observed in native tau-441, GSK3β-tau exhibits several deep, well-defined troughs, indicating the presence of distinct and stable conformational states (Fig. 3a). These states are also apparent in the top-down projection shown in Fig. S13. Continuous energy landscapes over 120 s for native tau-441 and GSK3β-tau also exhibit qualitative consistency with those observed in the 20-s intervals, with only minor variations attributable to the intrinsic heterogeneity of IDPs (Fig. S14).

### Disorder to order transition of the Sam68 N-terminal region

Our results demonstrate that NOTs enable the direct and prolonged observation of conformational dynamics in single, unmodified IDPs/IDRs, and allow reconstruction of their free-energy landscapes. To illustrate the broader utility of this approach in capturing changes in the structural order of IDP/IDRs upon binding at the single-molecule level, we investigated the RNA binding kinetics of Sam68, a system not previously studied using single-molecule methods.

Sam68 is an RNA-binding protein composed of three main regions: the N-terminal region (residues 1–96), the central STAR binding domain (residues 97–260), and the C-terminal region (residues 261–443)^66^. It binds to G8.5 RNA, a 40-nt RNA sequence, with a dissociation constant (*K*_d_) of ~12 nM determined from an electrophoretic mobility shift assay^67^. This affinity, however, is significantly reduced to around 36.1 µM when only the central STAR domain is present^68^, suggesting that the intrinsically disordered N- and C-terminal regions contribute to strong binding. Experiments later confirmed that both termini independently bind G8.5 RNA, with affinities of 1–10 µM for the N-terminal region and 30–70 µM for the C-terminal region^69^.

**Fig. 4:**
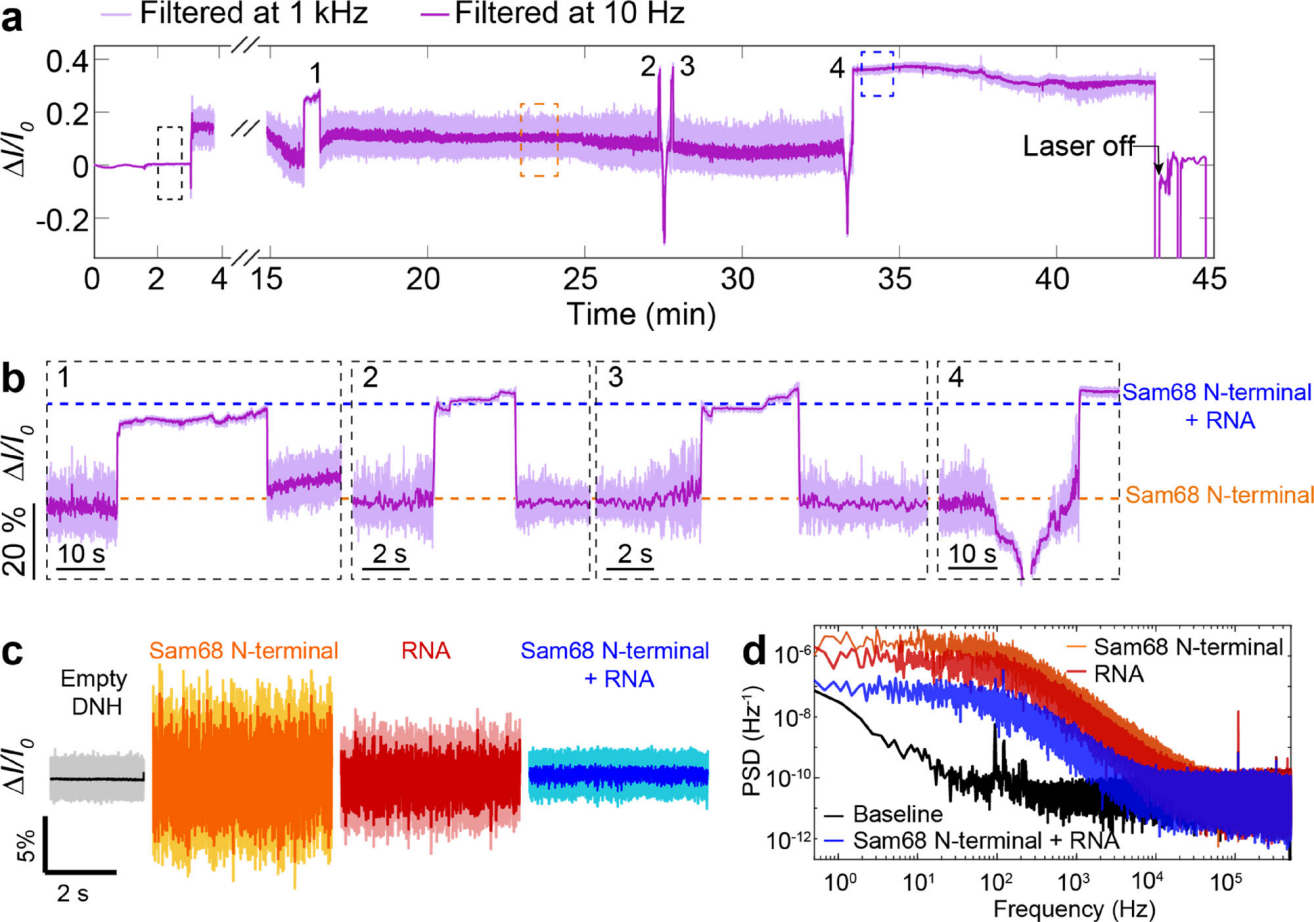
Binding of G8.5 RNA to the Sam68 N-terminal region. **a** Transmission trace of optical trapping of the Sam68 N-terminal region followed by binding and unbinding of G8.5 RNA. Data was filtered to 1 kHz (light purple) and 10 Hz (dark purple). **b** Four binding/unbinding events depicting the reduction in signal fluctuation of the Sam68 N-terminal region from unbound (orange) to RNA-bound (blue) states. **c** Zoomed segments from the dashed boxes in (**a**) depict the baseline (black), Sam68 N-terminal region before RNA binding (orange) and the formed Sam68-RNA complex after RNA binding (blue). The RNA trace was acquired separately, with the full trace shown in Fig. S16b. Data for traces displayed with raw data (1 MHz, light colours), and digitally filtered data (1 kHz, dark colours). **d** PSD plots of normalised traces for the baseline (black), RNA (red), Sam68 N-terminal region (orange) and the Sam68-RNA complex (blue) for comparison.

Here, we focus on the Sam68 N-terminal region and the structural changes which occur upon binding to G8.5 RNA. This RNA-binding activity is attributed to the two arginine/glycine (R/G) rich motifs (^45^RGGGGG^50^ and ^52^RGG^70^) on the Sam68 N-terminal region, and the adenosine/uracil (A/U) rich motif (^22^AUUAAAA^28^) in G8.5 RNA^70^ (full sequences are presented in Fig. S15a, b). We first trapped the N-terminal region of Sam68 for ~15 min before introducing 1 μM G8.5 RNA to monitor binding dynamics (Fig. 4a). Around 1 min after the arrival of G8.5 RNA at the trapping site (~16 min after trapping), a sharp increase in transmission occurs, indicative of a higher polarisability, due to greater hydrodynamic volume for the Sam68 N-terminal region-RNA bound state (referred to as the Sam68-RNA complex for the rest of this work) compared to the unbound protein. This increase is accompanied by a significant reduction in signal fluctuations (Fig. 4a, b), consistent with a transition to a more stable and ordered structure, representing the first direct observation of a disorder-to-order transition for the Sam68 N-terminal region from RNA binding. The Sam68-RNA complex remained associated for varying lengths of time, throughout the trap, lasting either ~30 s (Fig. 4b, inset 1) or ~2 s (Fig. 4b, insets 2 and 3), before dissociating and the RNA leaving the trapping region, characterised by the signal returning to a similar level as before binding, or remaining associated until turning the laser off (Fig. 4b, inset 4). As an additional control, to confirm whether G8.5 RNA alone would induce a noticeable change in the transmission signal, we also performed trapping experiments on G8.5 RNA independently of the Sam68 N-terminal region (Fig. S16). Trapping of G8.5 RNA produced a signal similar to trapping a protein molecule, with a noticeable signal change, and demonstrated a high level of dynamics, comparable to IDPs/IDRs, which adheres to our understanding of RNA being heterogeneous molecules^71^.

The differences between the Sam68 N-terminal region, G8.5 RNA, and the Sam68-RNA complex signal changes are most apparent in Fig. 4c. The Sam68 N-terminal region shows the highest level of signal fluctuations and, therefore dynamics, characteristic of IDPs/IDRs. Comparatively, G8.5 RNA alone also shows a high level of dynamics, whilst the Sam68-RNA complex demonstrates much lower dynamics than both, suggesting a more stable and ordered state.

Figure 4d compares the PSDs of the baseline (black), Sam68 N-terminal region (orange), G8.5 RNA (red), and Sam68-RNA complex (blue). The Sam68-RNA complex exhibits reduced signal fluctuations compared to the Sam68 N-terminal region alone at frequencies below 10 kHz (>100 μs), suggesting restricted dynamics and enhanced structural stability induced by RNA binding. A similar trend was observed in two additional trapping experiments where the Sam68 N-terminal region was more dynamic before binding to G8.5 RNA, where the subsequent Sam68-RNA complex becomes more stable (Figs. S17 and S18). These two additional traces are discussed in more detail in Supplementary Section SI-14.

This work demonstrates a key strategy for directly probing the conformational dynamics of single, label-free intrinsically disordered proteins (IDPs) and intrinsically disordered regions in structured proteins (IDRs) in solution. The trapping signals from nanoaperture optical tweezers (NOTs) reveal how IDP/IDR structural disorder manifests through distinct intensity fluctuations, energy states, and dynamic timescales, compared to globular proteins. At single-molecule resolution, we provide experimental evidence that phosphorylation of native tau-441 by GSK3β reduces conformational heterogeneity, with suppressed dynamics observed on the millisecond–second timescale. We further demonstrate the real-time observation of a disorder-to-order transition in the Sam68 N-terminal region upon RNA binding. These findings provide unique insights into the conformational dynamics of disordered proteins that are inaccessible to conventional single-molecule techniques, expanding the current experimental toolkit for studying protein disorder and its role in biological function and disease.

## Methods

### Fabrication of double nanohole structures in gold film

The double nanohole (DNH) structures used in this work were fabricated as previously described^31,32,37^. A 550 μm thick fused silica wafer was coated with a 30 nm silicon nitride layer using low-pressure chemical vapor deposition (LPCVD). Subsequently, a 5 nm titanium layer and a 100 nm gold layer were deposited using electron-beam evaporation at 190 °C. The wafers were diced into 10 × 10 mm chips for further use. Two nanoholes, each with a depth of 90 nm, a diameter of 160 nm and a centre-to-centre distance of 200 nm, were etched into the gold layer using a focused ion beam (FIB, Zeiss Crossbeam) with a gallium ion source. A rectangle of 3 nm in height was then etched to connect the edges of the two holes to form the DNH gap. The FIB was operated at 30 kV with a beam current of 1 pA.

### Double nanohole surface passivation

Double nanohole structures were passivated using polyethylene glycol methyl ether thiol (PEG-thiol, average MW 800 Da, 729108, Sigma Aldrich) as previously described^31,37^. The double nanohole samples were immersed in a solution comprised of 2 mM PEG-thiol in ethanol and left overnight (~18 h) before being rinsed thoroughly with ethanol and dried using an air gun. Solutions were freshly prepared before each use.

### Nanoaperture optical tweezers setup

Optical components were purchased from Thorlabs as previously described^31,32,37^. A half-wave plate adjusts the polarisation of the 852 nm laser (Thorlabs, FPL852) to be across the pointed edges of the gap between the two nanoholes (*y-*axis, Fig. 1a)^31,72^. The laser was collimated and expanded to 5 mm in diameter and then focused onto the DNH using a 100× objective (1.25 NA PLN100XO, Olympus). The laser power on the DNH was around 20 mW correlating to around 37 °C at the focusing point due to laser heating (Fig. S2). Light passing through the sample was collected with a 4× objective (0.1 NA PLN4XP, Olympus), then was focused onto an avalanche photodiode (APD120A/M, Thorlabs), which converted the light intensity to a voltage signal.

### Data acquisition

The avalanche photodiode (APD120A/M, Thorlabs) has a bandwidth of 50 MHz. However, considering the signal bandwidth of the system (~10 kHz) and to optimise the file size, the voltage signal was recorded at a sampling rate of 1 MHz using a data acquisition card (USB-6361, NI) controlled by a custom LabVIEW program. Based on the Nyquist frequency, this system provides a theoretical time resolution of 2 μs.

### Microfluidics system

Flow cells were printed using a FormLab 2 printer with Clear V4 resin at a resolution of 50 μm (FormLabs Inc, USA), as previously described^31,32,37^. Two-component silicone glue (Twinsil, Picodent, Germany) was used to seal the DNH structure within the flow cell with a 0.17 mm thick glass coverslip. A 50 μm thick piece of double-sided tape (Arcare 92712, Adhesive Research, Inc) was used to separate the DNH and glass coverslip, creating a chamber with a volume of 3.5 μL. Flow rate and flow direction were controlled using a syringe pump (Harvard Apparatus, US) through a 12-valve distributor (MUX Distributor, Elveflow, France).

### Minimising the effect of DNH geometry on the signal

Only traces from molecules trapped in DNHs with the same/similar geometries were used in our comparisons to minimise signal variations arising from differences in DNH geometry. Additionally, only traps obtained within 2 weeks from a sample's first use are used to reduce signal variations from degradation of the DNH.

### Protein and RNA preparation

Human haemoglobin (H7379, Sigma Aldrich), human native tau-441 (T0576, Sigma Aldrich), human GSK3β-tau-441 (SRP0689, Sigma Aldrich), bovine ribonuclease A (R5500, Sigma Aldrich), and bovine serum albumin (A8531, Sigma Aldrich) were prepared in a filtered buffer solution of 0.1 M bis-tris propane, 150 mM NaCl and 20% glycerol at pH 7.2. Bovine actin (A3653, Sigma Aldrich) was prepared in 0.1 M bis-tris propane, 0.2 mM CaCl_2_, 0.2 mM ATP and 20% glycerol at pH 7.2.

Human Sam68 N-terminal (amino acids 1–96) and C-terminal (amino acids 267–368) sequences were cloned and produced at the University of Leicester and comprised as previously described^69^ (sequence also available in Fig. S15a). G8.5 RNA sequence was bought from Dharmacon, Horizon Discovery and is the same as previously described^67^ (sequence also available in Fig. S15b). RNA and both protein solutions were prepared in a filtered solution containing 50 mM sodium phosphate and 150 mM NaCl at pH 6.8, whilst the RNA buffer also had RNase Inhibitor (AM2694, Thermo Fisher Scientific) added at 1 U/μl.

Proteins were aliquoted into 1 μM aliquots of 100 μL volume and immediately flash frozen in liquid nitrogen before being stored at −20 °C, except for GSK3β-tau, which was stored at −80 °C. The proteins were slowly thawed on wet ice on the day of use for an experiment.

### Data analysis

Custom MATLAB scripts were used to analyse all the data in this work.

### Data filtering

Raw data were filtered using a zero-phase Gaussian low-pass filter to the desired cut-off frequency by using the **filtfilt.m** function.

### Normalisation of optical transmission traces

We used normalised transmission intensity, Δ*I/I*_*0*_, to quantify the relative transmission change upon trapping a single protein. Since the optical signal was recorded by the avalanche photodiode as voltage (V), with Δ*I/I*_*0*_ calculated as Δ*I/I*_*0*_ = (*V* *–* *V*_*0*_)/ *V*_*0*_. For trapping traces shown in Figs. 2a–c, 4a, S4a, b, S9a–c, S16a–c, S17a and S18a, *V*_*0*_ is the mean value of the baseline, whilst for the trapped transmission traces (Figs. 2f–h, S8a–c, S17b, c and 18b, c), *V*_*0*_ corresponds to the mean value of the trace.

### Probability density function (PDF)

We filtered the 20-s trace with a cutoff frequency of 10 kHz and calculated the PDF by estimating the kernel density using the **ksdensity.m** function with 300 points. This cutoff frequency was chosen based on the PSD analysis (Fig. 4c, S8d, S17d and S18d), which shows that protein-induced signal variations remained distinguishable from empty DNH noise up to 10 kHz.

### Trace detrending

To allow well-assigned levels for step fitting, we removed the linear drift of the whole trace using the **detrend.m** function from MATLAB 2022b, as shown in Figs. S17a and S19.

#### Autocorrelation function and time constant (ACF)

We calculated the autocorrelation function from 5 s segments of raw traces using the MATLAB function *autocorr.m*. The resulting autocorrelation curves were fitted with exponential decay functions, with weighting applied according to the magnitude of the autocorrelation to reduce the influence of points at longer lag times. We initially applied a single-exponential fit to assess its adequacy; when this model failed to provide a good fit (i.e., *R*^2^ < 0.9 for the data points of first 50 ms), we then fit the curves with a double-exponential decay function instead (Figs. 1c, d and 2e-h).

### Deconvolution of PDF and energy landscapes

See details in Supplementary Section SI-11.

### Power spectral density (PSD)

To estimate the PSD of the time-domain signal across the trapping trace, we used the sampling frequency (*f*) and the signal vector (*XXX*). The frequency vector was taken over half of the frequency spectrum, from 0 to *f*/2 for the Nyquist frequency, using a linearly spaced grid with the number of data points (*N*)/2 in size. The power spectrum was then computed using the Fast Fourier Transform (FFT), and the squared magnitude was obtained using |FFT(*XXX*)|^2^ before normalisation by *N* multiplied by *f* as shown in Eq. 1:

1 $${{Pxx}}_{{\mathrm{temp}}}=\frac{{FFT}\left({XXX}\right)\times {conj}\left({FFT}\left({XXX}\right)\right)}{N\times f}$$

To remove negative frequency components and conserve the total power in the spectrum, the power spectrum was truncated to *N*/2 points and multiplied by 2 as shown in Eq. 2:

2 $${Pxx}=2\times {{Pxx}}_{{\rm{temp}}}\left(1:\left[\frac{{\rm{N}}}{2}\right]\right)$$

### Finite-difference time-domain simulations

The transmission of DNH structures was modelled based on finite-difference time-domain (FDTD) using commercial software (Lumerical, Ansys). See Tables S1 and S2 in Supplementary Section SI-3 for parameters.

### Volume versus transmission changes

The relationship between particle volume and the change in transmission (Fig. S3d) was simulated by placing a spherical particle (refractive index, $n$ = 2) with radii ranging from 2 to 6 nm in the centre of the DNH (*x* = 0, *y* = 0, *z* = 8) (Table S1).

### Particle distance versus transmission changes

The effect of particle distance from the SiN-Au interface (*z* = 0) (Fig. S3e) was simulated by measuring transmission changes of a spherical particle (refractive index, $n$ = 1.8, radius, *r* = 4 nm) at distances ranging from −200 to 10 nm.

### Laser heating simulation

The laser heating simulation is similar to that described previously^32^, with details listed in Supplementary Section SI-2.

## Supplementary information

IDP Paper SI

## Data Availability

The datasets generated and/or analysed during the current study are not publicly available due to the large size of the raw data, but are available from the corresponding author on reasonable request.

## Code availability

The codes used for data analysis are available from the corresponding author upon reasonable request.
