# Supplementary material for: Label-free optical observation of disordered-to-ordered transitions in single intrinsically disordered proteins
Source: NPJ Biosens. 2026 Jun 2;3(1):33. doi: 10.1038/s44328-026-00098-7 (PMC13246436; doi:10.1038/s44328-026-00098-7)
Supplement: Supplementary file 1 — IDP Paper SI [file 44328_2026_98_MOESM1_ESM.docx]

**Supplementary Information**

Label-free optical observation of disordered-to-ordered transitions in single intrinsically disordered proteins

Saaman Zargarbashi^1,2^, Cyril Dominguez^3,4^, Matthew Peters^5^, Arman Yousefi^1,6^, Sharon Munday^4^, Yanhong Wang^1^, Shreyasi Chatterjee^7^, Andrew J. Hudson^3^, Reuven Gordon^5^, Christopher J. Mellor^2^, Lei Xu^1^, Mohsen Rahmani^1,*^, and Cuifeng Ying^1,*^

^1^ Advanced Optics and Photonics Lab, Department of Engineering, School of Science and Technology, Nottingham Trent University, Nottingham, United Kingdom

^2^ School of Physics and Astronomy, University of Nottingham, Nottingham, United Kingdom

^3^ Leicester Institute for Structural and Chemical Biology, University of Leicester, Leicester, United Kingdom

^4^ Division of Molecular and Cell Biology, School of Biological and Biomedical Sciences, University of Leicester, Leicester, United Kingdom

^5^ Department of Electrical Engineering, University of Victoria, Victoria, British Columbia, Canada

^6^ Physical and Theoretical Chemistry Laboratory, Department of Chemistry, University of Oxford, United Kingdom

^7^ Department of Biochemistry, School of Science and Technology, Nottingham Trent University, Nottingham, United Kingdom

* Correspondence to: [cuifeng.ying@ntu.ac.uk](mailto:cuifeng.ying@ntu.ac.uk); [mohsen.rahmani@ntu.ac.uk](mailto:mohsen.rahmani@ntu.ac.uk)

SI-1 Nanoaperture optical tweezers setup


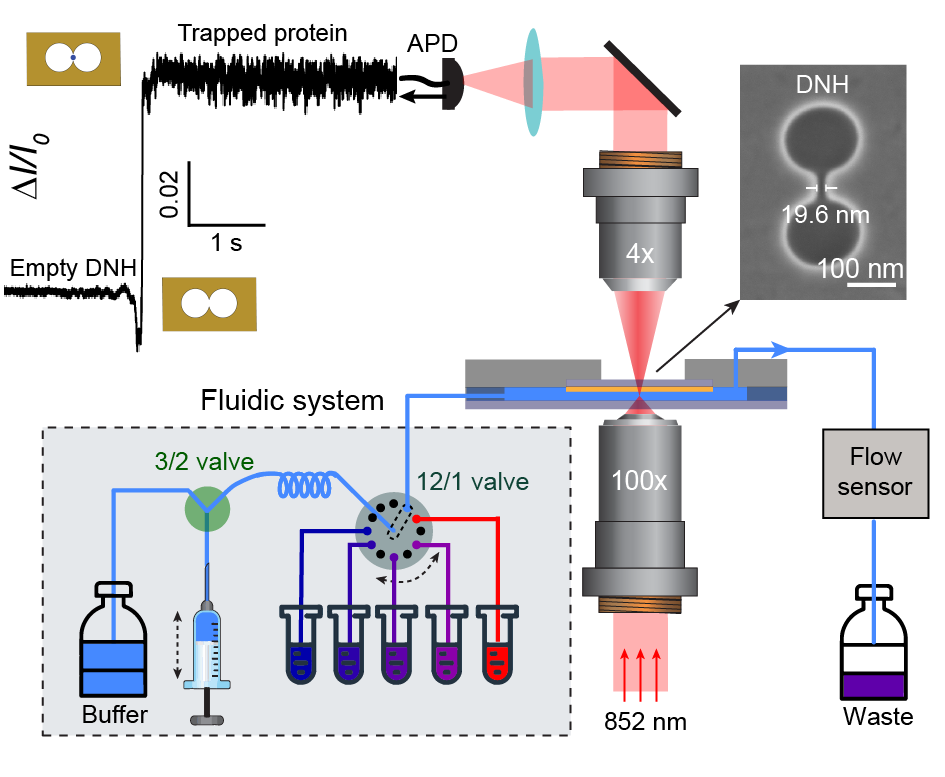


Fig. S1| Schematic of the nanoaperture optical tweezers setup. A Collimated laser at 852 nm is focused onto the double nanohole (DNH, with SEM image on the right) structure by a 100x objective (NA 1.25) to excite localised surface plasmon resonance (LSPR). Laser transmitted through the DNH is subsequently collected by a 4x objective (NA 0.1) and detected by an avalanche photodiode (APD) to record the transmission intensity over time. **Top left inset:** transmission intensity trace change upon trapping a protein. Increased fluctuations in the normalised transmission intensity (Δ*I/I_0_*) signify the trapping of a protein. **Bottom left inset:** an incorporated microfluidics system allows altering solution conditions whilst the protein is trapped. See materials and methods for details.

**SI-2 Laser-induced heating**

**Figure S2** shows the simulated laser heating profile of the DNH structure obtained through finite element simulations. We performed the simulation using the transient heat transfer model of COMSOL Multiphysics as described in previous work^1^. The laser was focused onto the gold layer with a spot size of 1.36 µm (measured). The absorption coefficient of water and gold at 852 nm were set to be 4.4372 m^-1^ and 7.87 × 10^7^ m^-1^, respectively.

**
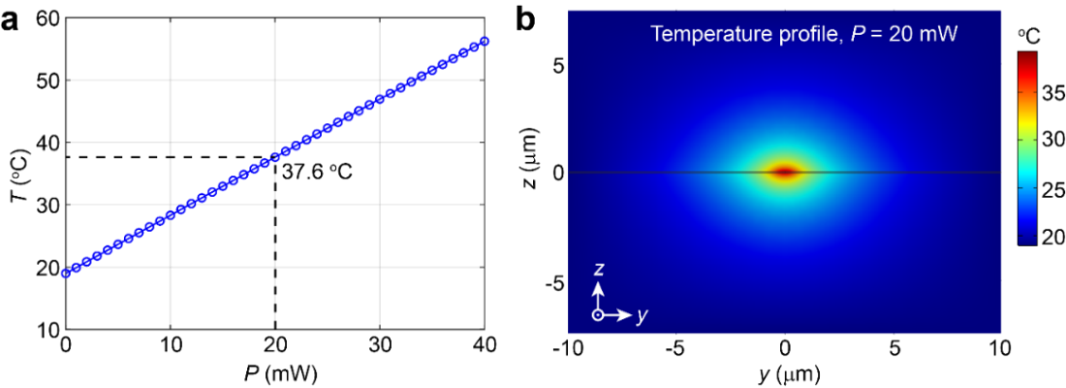
**

Fig. S2| Finite element simulation of laser heating. Simulated laser heating of the DNH when illuminated by a focused laser beam (852 nm) with a spot size of 1.36 μm (100×, NA 1.25). **a**, Linear relationship between laser power and temperature at the hotspot, with 20 mW marked by dashed line. **b**, Temperature profile of the laser induced heating effect at a laser power of 20 mW. The room temperature was set to 19 °C.

**SI-3 Finite-difference time-domain (FDTD) simulations**

To understand the relationship between transmission intensity changes, Δ*I/I_0_*, and the shape anisotropy and volume of the trapped particle, we conducted finite-difference time-domain (FDTD) simulations using commercial software (Lumerical, Ansys). **Figures. S3a–c** confirm the strong field enhancement localised in the DNH gap due to localised surface plasmon resonance (LSPR) excitation. Introduction of a spherical particle (edge positioned ~2.5 nm from the gold surface, **Fig. S3c**) further enhances the local field intensity, thereby stabilising the trapping of the particle.


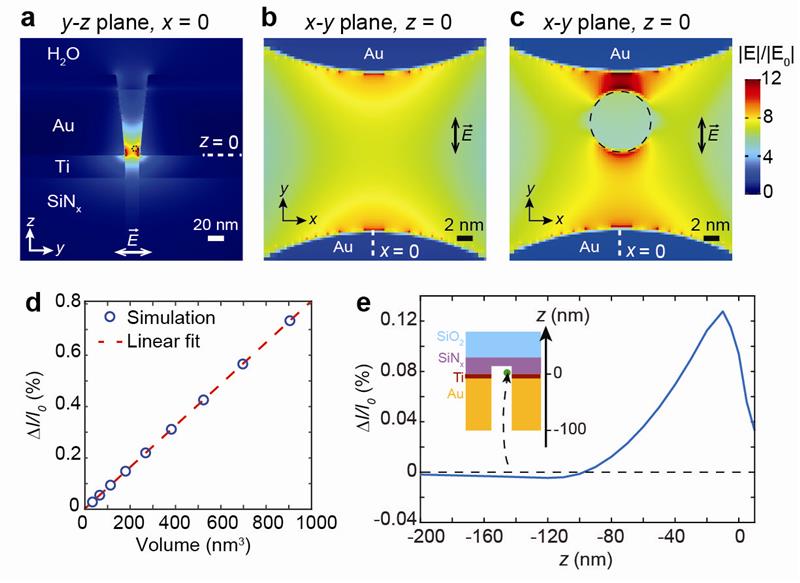


Fig. S3| **a**, Field distribution in an empty DNH on the *y-z* plane at *x* = 0. **b** and **c**, Zoomed field enhancement of an empty DNH (**b**) and a DNH containing a spherical particle (**c**) (refractive index, $n$ = 2, radius, *r* = 4 nm) at *z* = 0. For each simulation the incident laser (852 nm ± 10 nm) is polarised along the *y*-axis. **d,** Linear relationship between the change in normalised transmission intensity, Δ*I/I_0_*, and volume of spherical particles (*n* = 2) within the DNH gap, as indicated by the dashed circle in **a**, along with the linear fit (red dashed line). Spheres with 9 different radii, positioned with an edge-to-edge distance of 2.5 nm from the DNH, were simulated with parameters detailed in Table S1. **e**, Simulated change in transmission intensity based on the distance of a spherical particle (refractive index, $n$ = 1.8, radius, *r* = 3 nm) to the SiN-Au interface (*z* = 0) of a DNH with a gap size of 15 nm.

**Figure S3d** presents the simulated effects of particle volume on Δ*I/I_0_*. The presence of the nanoparticle alters the refractive index within the DNH gap, leading to changes in transmission intensity, Δ*I/I_0_*. The refractive index of a nanoparticle depends on its polarisability, which is associated with volume, shape anisotropy, and dielectric constant^2^. The FDTD simulations reveal a linear relationship between Δ*I/I_0_* and spherical particle volume (**Fig. S3d,** the particle parameters used are listed in **Table S1**). This relationship suggests that a larger protein produces a greater Δ*I/I_0_* signal when trapped in the same DNH structure. The influence of ellipsoidal geometry on Δ*I/I_0_* is complex, as it depends on both the aspect ratio and the orientation relative to the DNH. For spherical particles (aspect ratio = 1), no orientation-dependent signal changes occur, therefore, we expect stable Δ*I/I_0_* with non-system fluctuations primarily arising from translational diffusion within the trapping well. For non-spherical geometries (aspect ratio ≠ 1), depending on the orientation, Δ*I/I_0_* can either increase or decrease relative to an equivalent volume sphere (**Fig. 1e**). While rotational diffusion would theoretically cause larger Δ*I/I_0_* fluctuations for non-spherical particles, we consistently observe greater, yet stable, transmission changes for globular proteins in extended conformations compared to compact states^3–5^. The absence of increased fluctuations in Δ*I/I_0_* might be attributed to the optimal alignment of ellipsoidal particles, as the self-induced back action (SIBA) trapping mechanism preferentially stabilises orientations that maximise Δ*I/I_0_*. **Figure S3e** illustrates the influence of the distance of the trapped particle from the SiN-Au interface within a DNH gap on Δ*I/I_0_*. The dip before the trapping events shown throughout this work arise from transmission changes as the trapped particle occupies different vertical (z) positions (inset, Fig. S3e) and has been described previously^5^.

| **Table S1.** Particle parameters used for Lumerical simulation in Fig. S3d. | |
| --- | --- |
| Radius (nm) | Volume (nm^3^) |
| 2.0 | 33.51 |
| 2.5 | 65.45 |
| 3.0 | 113.10 |
| 3.5 | 179.59 |
| 4.0 | 268.08 |
| 4.5 | 381.70 |
| 5.0 | 523.60 |
| 5.5 | 696.91 |
| 6.0 | 904.78 |

| Table S2. Parameters for ellipsoid dimensions for Lumerical simulation in Fig. 1e. | | | |
| --- | --- | --- | --- |
| *A* (nm) | *B* (nm) | Aspect ratio (*A*/*B*) | Volume (nm^3^) |
| 2.4 | 14.6 | 0.164 | 267.86 |
| 3.0 | 13.0 | 0.231 | 265.46 |
| 3.6 | 12.0 | 0.3 | 271.43 |
| 4.8 | 10.2 | 0.471 | 261.48 |
| 6.6 | 8.8 | 0.75 | 267.61 |
| 8.0 | 8.0 | 1.0 | 268.08 |
| 8.8 | 7.6 | 1.158 | 266.14 |
| 9.2 | 7.4 | 1.243 | 263.78 |
| 11.2 | 6.8 | 1.64 | 271.10 |
| 12.8 | 6.4 | 2.0 | 274.52 |

**SI-4 Other trapping traces of IDPs/IDRs and globular proteins of similar molecular weight**


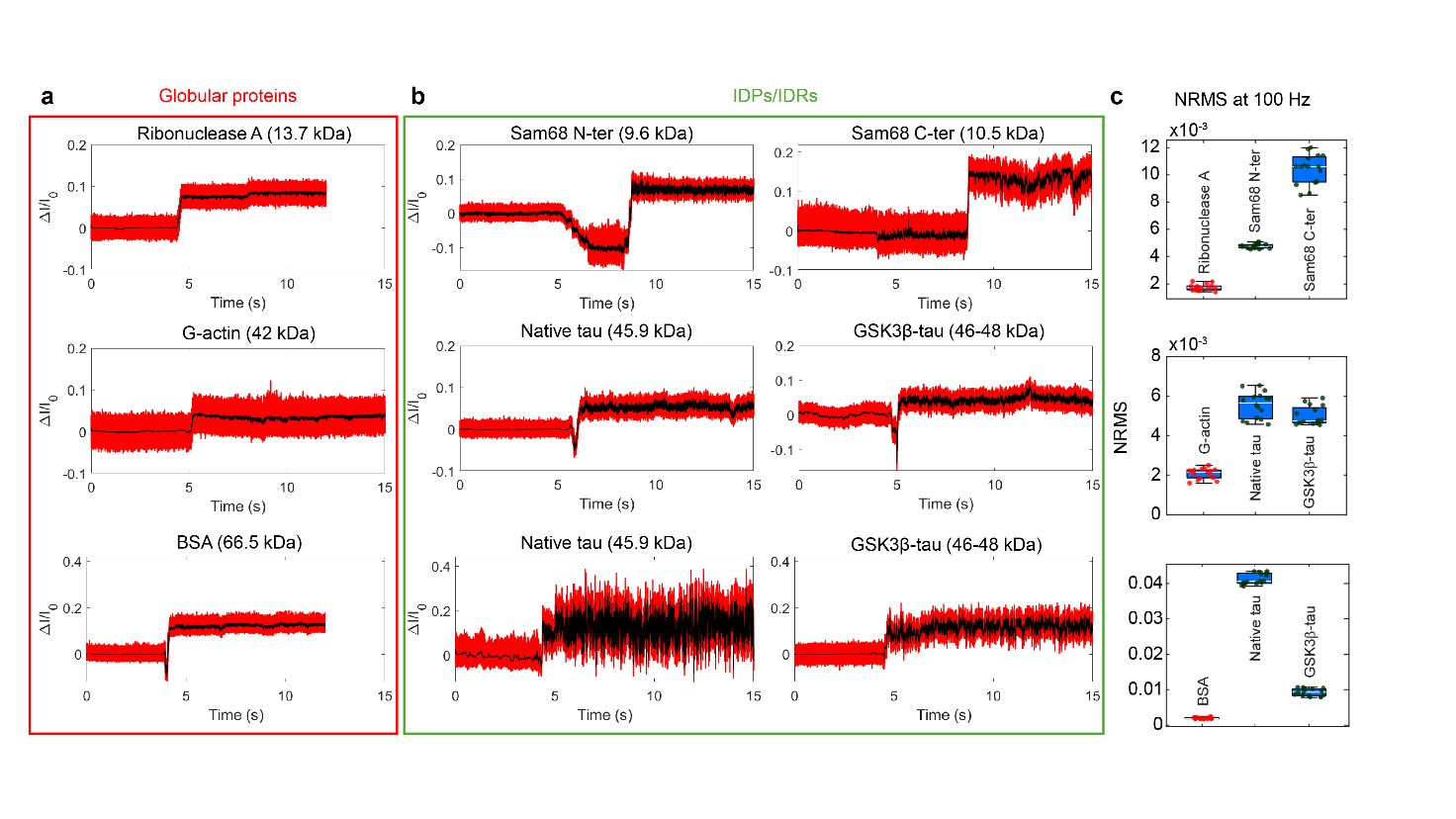


Fig. S4| Comparison of trapping traces for globular proteins (**a**, red box) and IDP/IDRs (**b**, green box), along with **c**, a boxplot of normalised root mean square (NRMS) values derived from transmission intensity traces. Trapping traces shown are raw (red) and digitally filtered at 1 kHz (black). The NRMS was calculated from traces filtered at 100 Hz to assess low-frequency fluctuations, using a 5-second sliding window shifted in 1-second increments. At similar molecular weights, IDP/IDRs exhibit greater signal fluctuations during trapping, reflecting their increased structural flexibility compared to globular proteins. Traces were acquired using different double-nanohole (DNH) structures; only those with similar Δ*I/I_0_* values upon trapping are compared here.

**SI-5 Extended protein conformations increase the local refractive index**

The refractive index ($n$) can be described by the Lorentz-Lorenz equation^6^ for a solution containing $N$ particles, each with a polarisability of $\alpha$, the relationship is given by:

$\frac{n^{2}-1}{n^{2}+2}=\frac{4\pi}{3}N\alpha$ (S1)

Within the DNH sensing volume (field enhanced zone), containing both solution and protein molecules, **Eq. S1** becomes:

$\frac{n^{2}-1}{n^{2}+2}=\frac{4\pi}{3}(N_{p}\alpha_{p}+{N_{w}\alpha_{w}+N}_{w}^{'}\alpha_{w}^{'})$ (S2)

Where, $N_{p}$ and $\alpha_{p}$ are the number and polarisability of amino acids within the protein; $N_{w}$ and $\alpha_{w}$ are the number and polarisability of water molecules in the bulk volume; and $N_{w}^{'}$ and $\alpha_{w}^{'}$ are the number and polarisability of water molecules in protein cavities or the near-surface layer, comprising the hydration shell of the protein.

The hydration shell exhibits a higher average water density than bulk water due to the electrostatic fields generated by protein atoms, which induce a preferential alignment of water dipoles. When protein molecules unfold­ ­­from compact to loosely extended conformations, the number of water molecules in the hydration shell ($N_{w}^{'}$) increases as water molecules penetrate newly accessible regions^7,8^. Meanwhile, the number of bulk water molecules ($N_{w}$) and the intrinsic properties of the amino acids remain unchanged. This increase in water density enhances the local refractive index within the sensing volume considering the relationship shown in **Eq. S2**.

For globular proteins, extended conformations yield higher Δ*I/I₀* signals compared to their folded states^5,9^. This effect arises from both local refractive index changes (mentioned above) and shape anisotropy at a specific orientation, where the SIBA trapping mechanism induces bias in the protein towards the orientation that maximises transmission intensity changes. For IDPs, which continuously sample compact and extended conformations on sub-microsecond-to-second timescales^10^, the rapid changes in local refractive index due to hydration dynamics lead to significant signal variability. Although different orientations can yield orientation-dependent Δ*I/I_0_* variations as illustrated in **Fig. 1e**, they appear in a fast timescale (< 100 ns). In this work, our measurements analyse signal fluctuations on timescales longer than 100 μs (i.e., < 10 kHz as shown in Fig. 1c), which are orders of magnitude slower than the timescale for rotational diffusion.

**SI-6 Scanning electron microscopy (SEM) images of representative DNH structures and effect of steric limitations on accessible protein conformations**


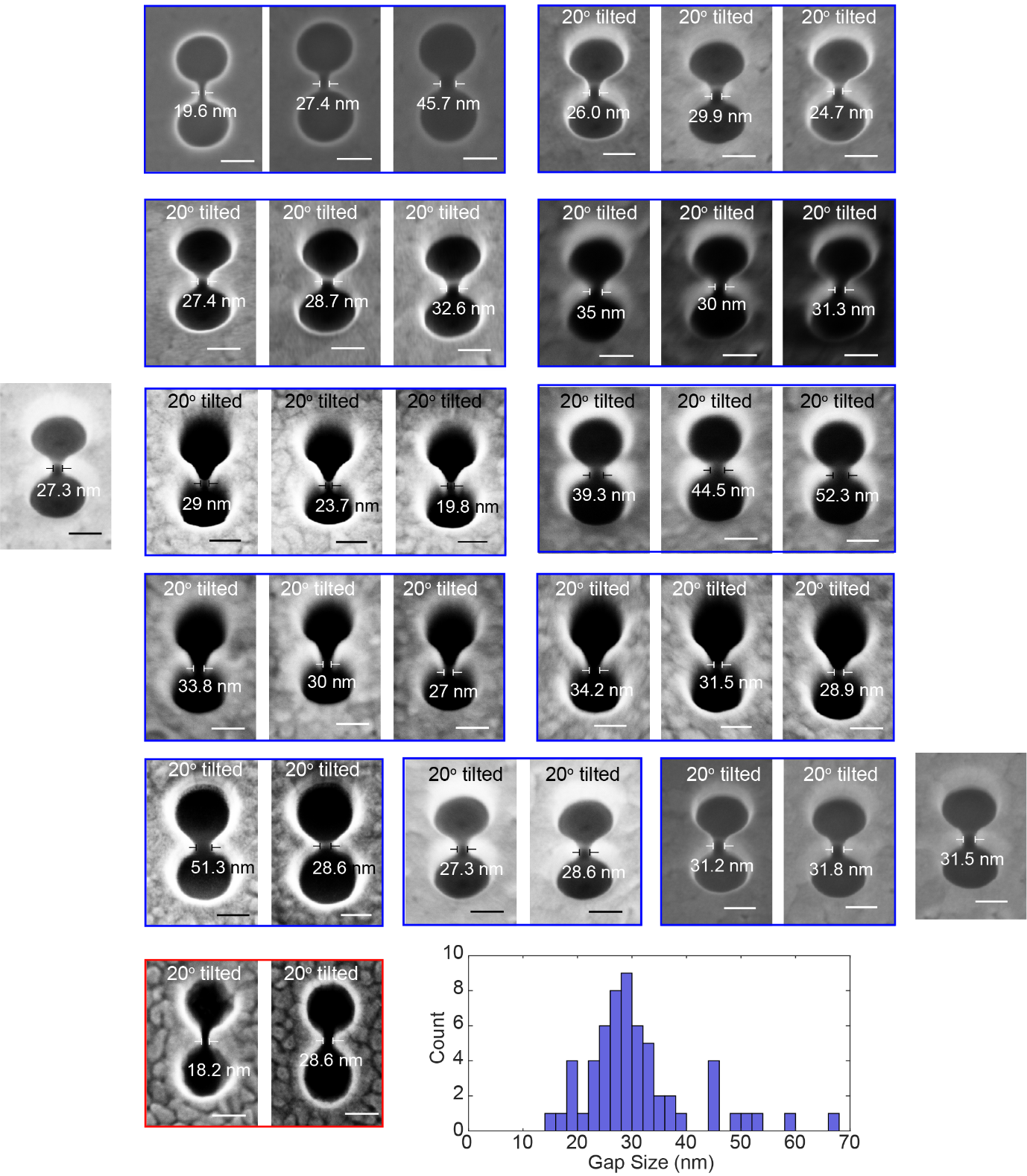


Fig. S5| Representative SEM images demonstrating variations in DNH geometries using the same fabrication parameters. Structures from the same chips (blue / red boxes) show smaller gap size deviations compared to gap size differences across chips. DNHs fabricated by a third party (red box) were employed to confirm experimental reproducibility. **Bottom right:** gap size distribution histogram revealing gap sizes predominantly lie within 25-35 nm (*n* = 61 structures). Note: SEM imaging induces irreversible damage, making the imaged structures no longer suitable for trapping experiments. Therefore, all trapping experiments were conducted using non-imaged structures, assuming comparable geometries as DNHs from the same fabrication batch.

Steric limitations may impact the accessible conformations of the proteins with the trap. The largest size potential in this study, tau-441, has an average hydrodynamic radius (*R*_h_) of ~5.4 nm and radius of gyration (*R*_g_) of ~6.5 nm, with a maximal size of ~12 nm^11,12^. The majority DNHs used in this work, with a average gap size ~28 nm (Fig. S5), should impose little, if any, steric limitations on the tau proteins trapped. Smaller gap sizes however may prevent proteins exploring their most elongated conformation. Consistent with this, we observed smaller signal fluctuations (Fig. 2f) using a DNH with a gap size of ~18 nm (red box in Fig. S5), yet comparisons using the same DNH structure confirm that GSK3β-tau remains more compact and ordered. For the other proteins with smaller *R*_h_ and *R*_g_, including BSA or smaller IDPs/IDRs, we do not expect steric limitations.

**SI-7 Representative trapping trace showing protein adhesion within the DNH and likelihood of protein sticking**

Figure S6 illustrates a representative sticking event, where the trapped protein molecule is not released upon turning the laser off. Table S3 further lists the number of sticking events among the total number of trapping events we obtained.

The IDPs/IDRs demonstrate a higher probability of sticking in the trap (≥ 33%) compared to globular proteins (≤ 21%). Notably, there is a decreased sticking frequency for GSK3β-tau compared to native tau-441, despite being the same protein. This not only supports the conclusion that GSK3β-tau is more globular than native tau, but also follows the trend that IDPs/IDRs have a higher propensity to stick to the DNH compared to more globular proteins, likely due to their high conformational flexibility. An additional interesting observation is the significantly higher frequency of native tau-441 to stick than other IDPs (≥ 13.8%), even compared to the Sam68 N-terminal region, which we have shown to be highly dynamic (Figs 4, S17–S18). One possible reason for this difference may be due to the size difference of the two proteins (45.9 kDa for tau-441 vs 9.6 kDa for the Sam68 N-terminal region). As tau-441 is much larger, it may be more likely to come into close proximity to the walls of the DNH, increasing the likelihood of it sticking despite the PEG-thiol coating.

**
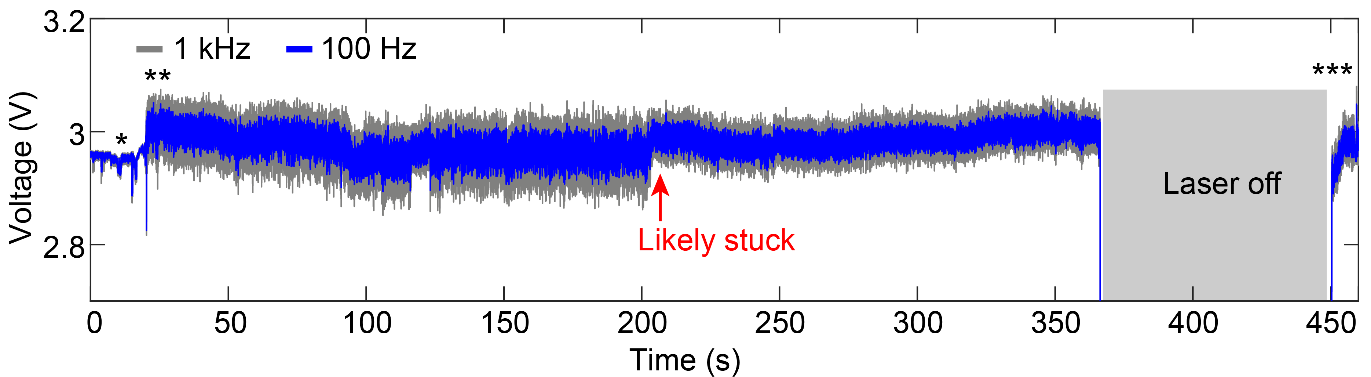
**

Fig. S6| Example trapping trace of GSK3β-tau showing protein adhesion in the DNH gap and does not release after the laser is turned off. Trace was recorded at 1 MHz and is shown filtered to 1 kHz (grey) and 100 Hz (blue). We hypothesise that the protein was stuck at the time indicated by the red arrow, as the reduction in signal fluctuation suggests restricted protein motion, likely due to adhesion to the gold surface. Asterisks indicate the following events: (*): baseline before trapping, (**): trapping event, (***): protein still stuck after turning the laser off.

| Table S3. Number of traps vs traps that would not release upon turning the laser off | | | |
| --- | --- | --- | --- |
| Molecule trapped | Number trapped | Number stuck | Stuck vs total (%) |
| Native tau-441 | 26 | 14 | 53.8 |
| GSK3β-tau | 21 | 7 | 33.3 |
| Haemoglobin | 24 | 5 | 20.8 |
| Sam68 N-terminal region | 19 | 7 | 36.8 |
| Sam68 C-terminal region | 5 | 2 | 40 |
| Other globular proteins | 6 | 1 | 16.6 |
| G8.5 RNA | 3 | 0 | 0 |

**SI-****8** Effect of GSK3β induced phosphorylation on tau-441 conformational dynamics

Post-translational modifications, such as phosphorylation, are of particular interest due to their role in regulating protein function and stability, as well as their contribution to pathological conformational changes in proteins when dysregulated. In tau, phosphorylation regulates its physiological role in microtubule binding *in vivo*^13^, while hyperphosphorylation is known to promote aggregation and pathogenic conformations associated with Alzheimer’s disease^14^.

Native tau-441 contains around 85 putative phosphorylation sites across serine (Ser), threonine (Thr), and tyrosine residues^15^. GSK3β is capable of phosphorylating up to 42 Ser/Thr residues, with site specificity dependent on the presence of a pre-phosphorylated (primed) motif. GSK3β preferentially targets sequences containing Ser/Thr-X-X-X-pSer/pThr and is classified as a proline-directed kinase^16,17^, although GSK3β can also phosphorylate unprimed sites^16^.

The structural effects of phosphorylation on IDPs depend on both the number and location of modified residues^18^, influencing overall disorder, compaction, and the conformational ensemble. The global conformation of an IDP is influenced by its proline content and net charge, with compaction typically being observed as either decreases^19^. Native tau-441 has a theoretical isoelectric point (pI) of 8.24 (**Fig. S7**), and so will present a net positive charge at physiological pH values. As such, by introducing negatively charged phosphate groups via phosphorylation, electrostatic interactions can drive compaction within native tau-441^20^.

Previous studies demonstrate that the conformational response of tau-441 to phosphorylation is highly context-dependent. For example, phosphorylation at S202 and T205^21^, or pseudo-phosphorylation at S199E, S202E, and T205E or at S396E and S404E^22^, has been associated with reduced compaction. Conversely, the combination of these pseudo-phosphorylation sites (S199E, S202E, T205E, S396E, S404E) induced global compaction, which was further increased upon the addition of T212E and S214E^22^. It is important to note that phosphorylated Ser and Thr residues typically present a double charge at physiological pH, than glutamic or aspartic acid (potential -2 vs -1)^23^ altering their effects on tau structure.

Our experimental data suggests that phosphorylation of native tau-441 by GSK3β leads to increased conformational order and compaction, as observed in the more defined and stable states sampled by GSK3β-tau compared to native tau-441 (**Figs. 2, 3, and S8**). Two primary mechanisms may underlie this behaviour. First, phosphorylation reduces the net charge of the positively charged tau, promoting compaction. Likely phosphorylation sites for isolated GSK3β include T175, T181, T205, T231, located in the proline rich domain, and S396, S400, and S404, in the C-terminal^24–26^ (**Fig. S7**). Although the exact phosphorylation pattern in our commercially obtained GSK3β-tau was not provided, literature suggests that T231 is a key GSK3β phosphorylation site and may be essential for subsequent phosphorylation events, particularly at the C-terminal following local conformational rearrangement at the N-terminal^27^. Second, phosphorylation may induce secondary structure transitions. Residues within the proline-rich domain (I151-Q244) are prone to adopt transient polyproline type II (PPII) helices^28^. Phosphorylation at T231 has been shown to promote α-helical structure formation^29^, which are more compact than PPII helices (5.4 Å/turn vs 9.3 Å/turn)^30^. The presence of a stabilising salt bridge between phosphorylated T231 and R230 has also been reported, further increasing conformational order^29^. Similarly, phosphorylation at S396 and S404 may permit salt bridge formation with local residues such as K395 or R406, leading to increased structural stability.

It is also important to consider the effect of the charge changes from phosphorylation on protein surface interactions and hydration, as these effects will impact the observed signal. As noted above, phosphorylation of tau-441 will reduce its net charge towards neutral or negative. Although we coat the DNHs with PEG-thiol, which exhibits a net neutral charge which minimises interactions and limits the maximum surface proximity of the protein to the DNH^31^, we acknowledge that such effects may still be present. The gold surface is negatively charged, and so GSK3β-tau will exhibit either weaker or opposing electrostatic interactions with the DNH surface compared to native tau-441. This could induce preferential orientations if these are electrostatically driven, and the repulsion between the protein and surface could induce the compacted state of GSK3β-tau observed here. We do not believe electrostatic effects to play a significant role in the signal based on three experiments where trapped G8.5 RNA, a charged molecule with a net negative charge shown in Fig. S16, which we then compare to the Sam68 N-terminal IDR, with a net positive charge (pI ≈ 12.08, Figs. 4c and S18c). Despite their opposing charges, G8.5 RNA presents dynamics at a comparable level to the Sam68 N-terminal region (Figs. 4c-d and S18c-d). A greater number of exposed charge groups is known to cause greater restriction in the hydration water around IDPs^32^. This has been shown to reduce the radius of hydration in IDPs, which may contribute to the more compact state of GSK3β-tau compared to native tau-441.


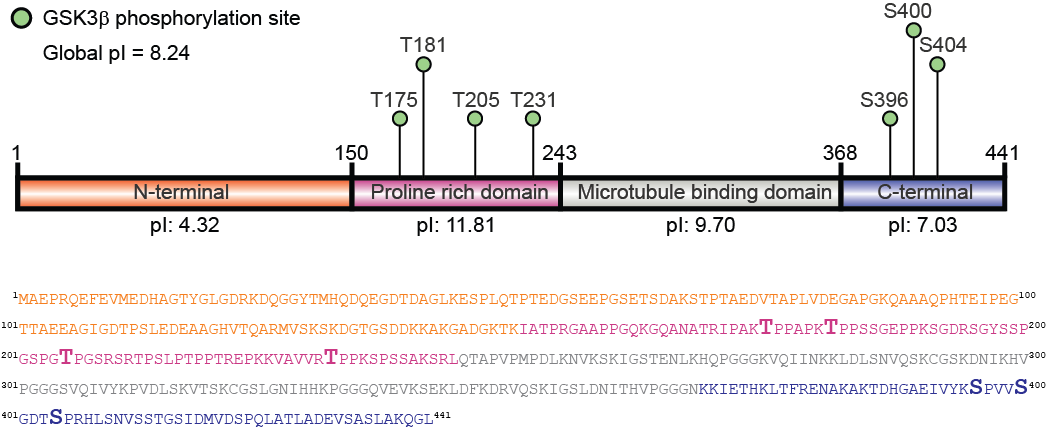


Fig. S7| Sequence and information for native tau-441. The four domain sequences in native tau-441, along with theoretical isoelectric points (pI). The seven assumed potential phosphorylation sites for GSK3β-tau within this study are labelled (green circles). The illustration was made using IBS^33^. Amino acid residues are coloured according to their relevant domain, and assumed GSK3β phosphorylation sites are listed in bold.

**SI-9** Trapping traces and PSDs for native tau-441 and GSK3β-tau


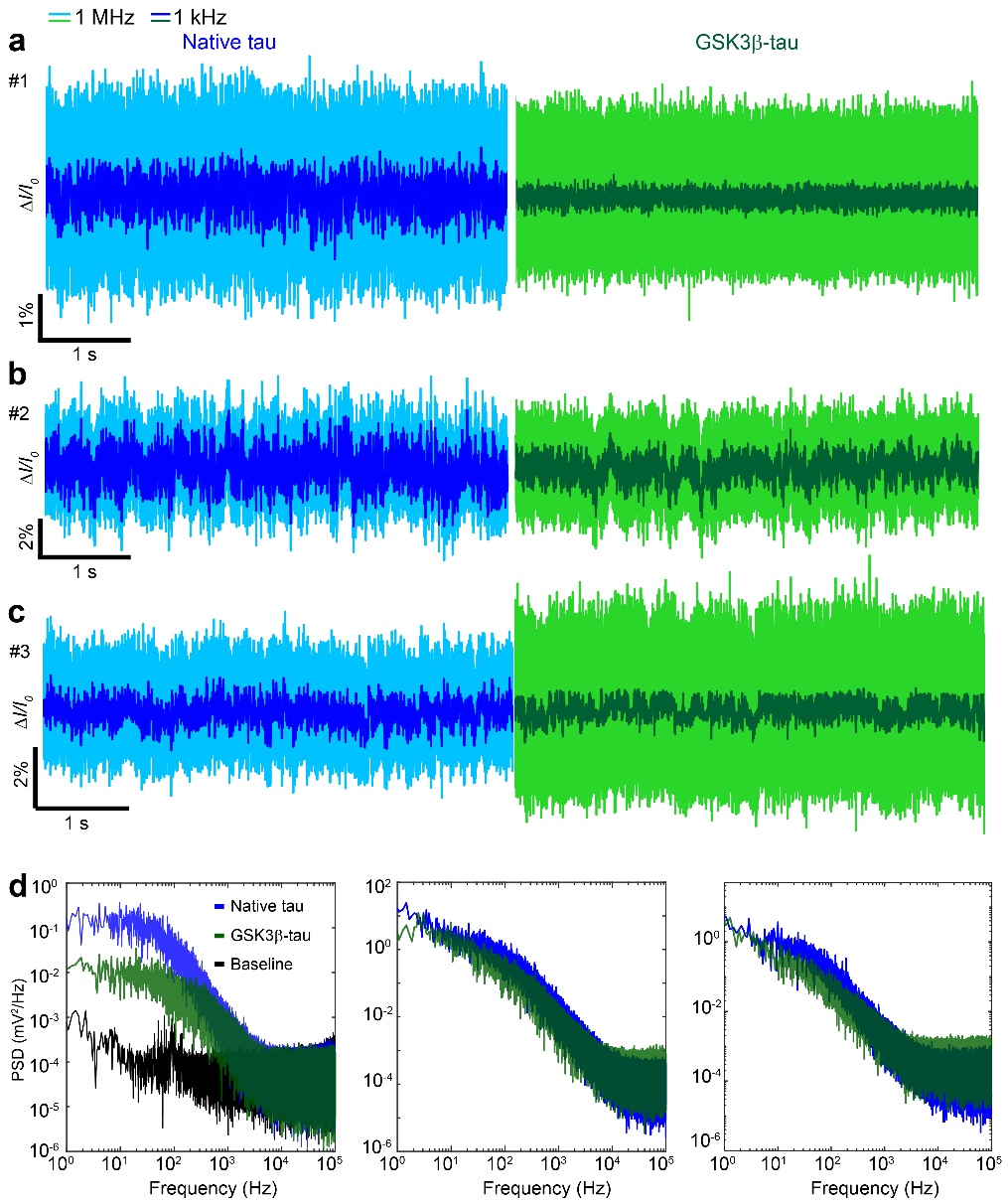


Fig. S8| **a-c** Five-second transmission intensity traces for three replicate datasets, comparing native tau-441 (blue) and GSK3β-tau (green), shown as raw (1 MHz; light colours) and digitally filtered (1 kHz; dark colours). **d**, Corresponding PSD plots for the 20-s traces partially shown in panels (a–c), indicating that native tau consistently exhibits higher signal power within frequencies below 1 kHz.

**SI-10 Raw trapping traces of haemoglobin, GSK3β-tau and native tau-441**

**
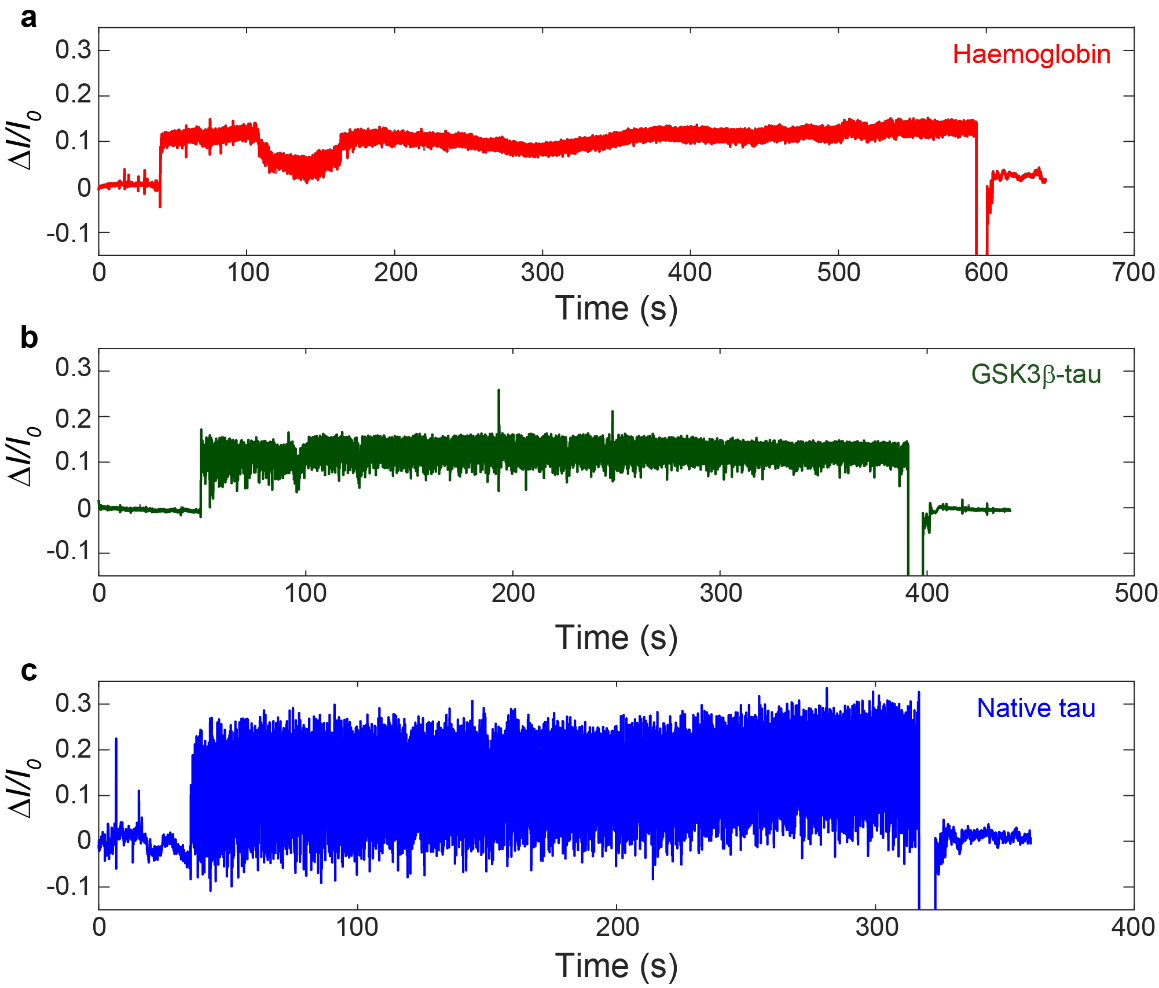
**

Fig. S9| Raw trapping traces of data shown in Fig. 2 for **a**, haemoglobin, **b**, GSK3β-tau and **c**, native tau-441. Data is shown filtered to 1 kHz.

**SI-11** Deconvolution of PDFs and calculation of energy landscapes

All probability density functions (PDFs) used for energy landscapes in the main text (shaded area of **Figs. 3a and 3c)** were calculated from 10 kHz filtered 20-second traces. Within the 10 kHz bandwidth, protein-induced signal variations are distinguishable from an empty DNH, demonstrated by the PSD in **Fig. S8d**. We deconvoluted these PDFs using a Gaussian point spread function (PSF) via the iterative Lucy-Richardson algorithm (deconvlucy.m), with the PSF as root mean standard deviation (RMSD) of 1-second of haemoglobin trapping.

We obtained the 2D energy landscapes by taking the negative logarithm of the deconvoluted PDF, as described previously^5^.

$E(\frac{\Delta I}{I_{0}})={-k}_{B}T\cdot ln[PDF\left( \frac{\Delta I}{I_{0}} \right)]$ (S3)

where *k_B_* is the Boltzmann constant, *T* is the temperature, and $\frac{\Delta I}{I_{0}}$ is the normalised transmission change.

To visualise multiple conformations, we converted 2D energy landscapes to 3D. To do so, we first fitted the deconvoluted PDF with a multi-peak Gaussian function,

$G\left( x \right)= \sum_{i=1}^{n} A_{i}e^{-2(\frac{x-\mu_{i}}{\sigma_{i}})}$ (S4)

where $A_{i}$, $\mu_{i}$ and $\sigma_{i}$ are the amplitude, peak location and standard deviation of the *i*-th Gaussian peak, respectively. The dashed curve in **Figure S10a** illustrates the multi-peak Gaussian fit to the deconvoluted PDF of GSK3β-tau (also presented in **Figure 3a**), with each peak displayed in different colours. Here, the PDF was intentionally overfitted to maximise accuracy in reconstructing the overall profile, as the fitting parameters are not intended to carry physical meaning.

We then converted the fitted Gaussian function to the energy landscape using

$U\left( x \right)={-k}_{B}T\cdot ln[G\left( x \right)]$ (S5)

where, the troughs in the energy landscapes correspond to the peaks in the PDFs. **Figure S10b** shows that the fitted energy landscape (black dashed curve) closely matches the measured curve (green curve).


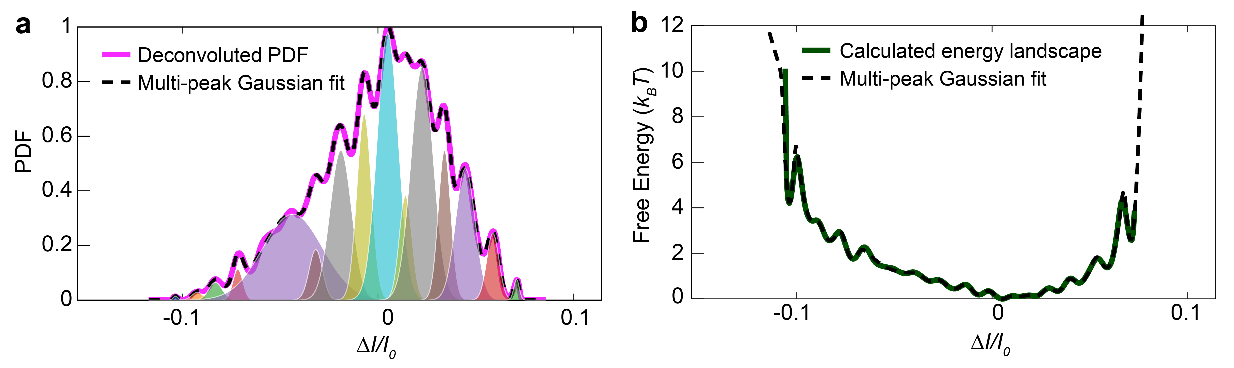


Fig. S10| Multi-peak Gaussian fit to **a,** PDF and **b**, conversion to 2D energy landscape. Colour-shaded regions in **a** represent individual fitted peaks. Values for Δ*I/I_0_* were taken over 20 s and digitally filtered to 10 kHz.

For 3D conversion, we mapped the peaks to a polar axis with radius *R* defined as half the distance between first and last peak locations, $R= \frac{{(\mu}_{N}- \mu_{1})}{2}$. The angular coordinate $\theta_{i}$ for the *i*-th peak was distributed between [0.1π, 0.9 π] based on peak position:

$\theta_{i}=0.9\pi\left( 1- \frac{\mu_{i}- \mu_{1}}{\mu_{N}- \mu_{1}} \right)+0.1\pi$ (S6)

This angular distribution ensures that different conformations are represented according to their relative position in 2D. The radial coordinate$r_{i}$is scaled by the peak amplitude:

$r_{i} =\frac{\max\left( A_{i} \right)-A_{i}}{\max\left( A_{i} \right)-\min\left( A_{i} \right)}R$ (S7)

**Eq. S7** ensures predominant peaks are located near the centre of the 3D plot. **Figure S11** shows peak locations on a polar plot.


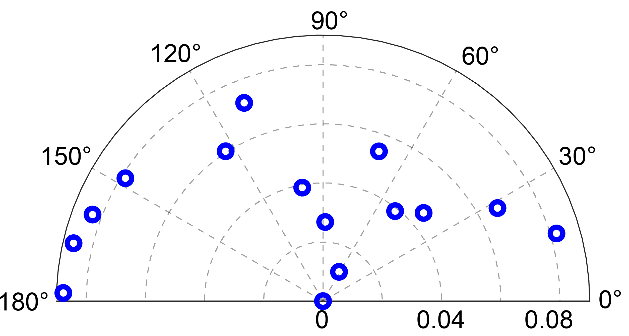


Fig. S11| Polar plot of peak locations, with each blue circle representing a fitted peak.

With the peaks being mapped onto a 2D surface, we converted the fitted PDF to a 3D multi-peak Gaussian function, using the peak locations$\left( \mu_{xi}, \mu_{yi} \right)=(r_{i}, \theta_{i})$, amplitudes $A_{i}$ and the standard deviations $\sigma_{i}$. Finally, the 3D energy landscapes (**Figs. 3b, S12, and S13**) were obtained by taking the negative logarithm of the 3D PDFs, with red and blue circles marking fitted troughs.


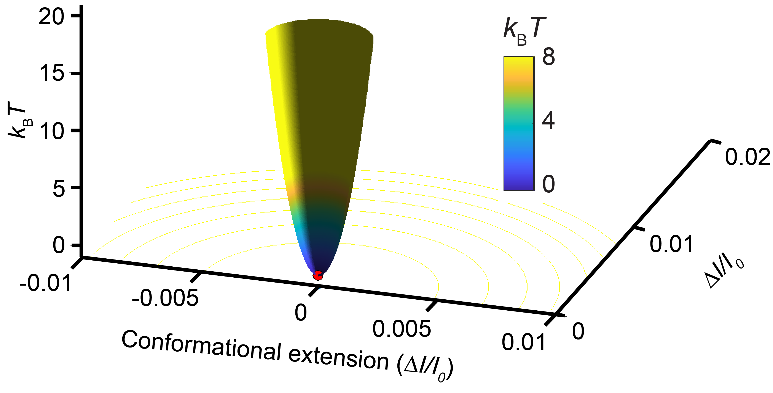


Fig. S12| 3D energy landscape of haemoglobin plotted using the deconvoluted PDF in Figure 3a. Funnel shape with a large energy barrier is representative of a typical globular protein with one dominant stable state. A small conformational extension range indicates little change in global shape.


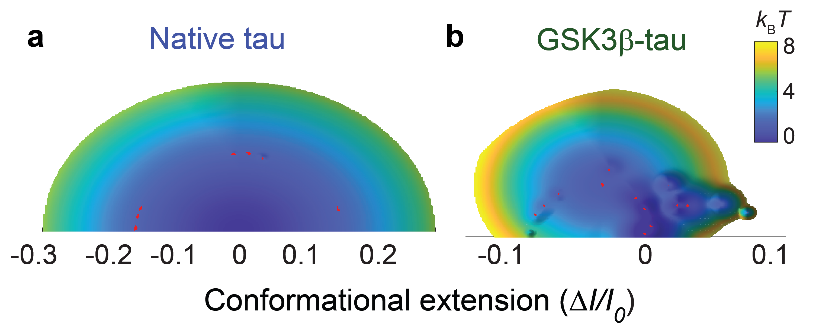


Fig. S13| Top-down view of 3D landscapes shown in Fig. 3b for **a**, native tau-441 and **b**, GSK3β-tau. GSK3β-tau displays more troughs than native tau-441, indicating new conformational states not sampled by the unphosphorylated variant.


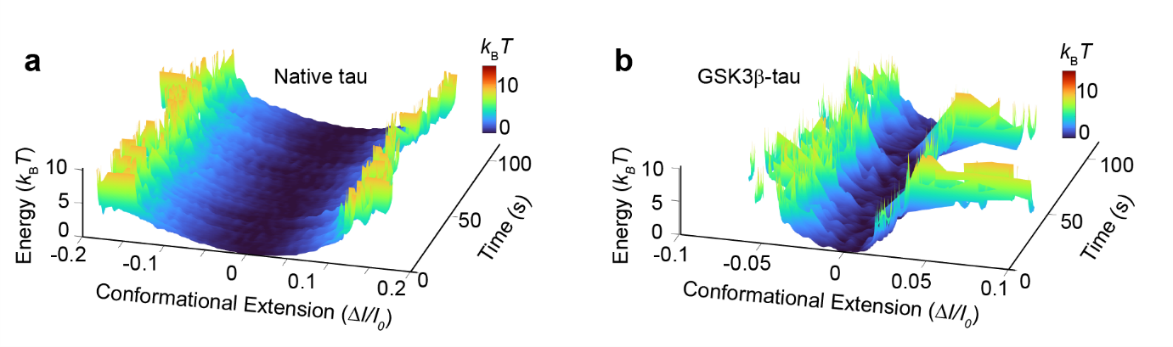


Fig. S14| Continuous 3D energy landscapes of IDPs over 120 s. **a**, Native tau-441 and **b**, GSK3β-tau. The energy landscapes were calculated from 1 kHz filtered data using a 5-second window sliding with 100-ms step increments.

**
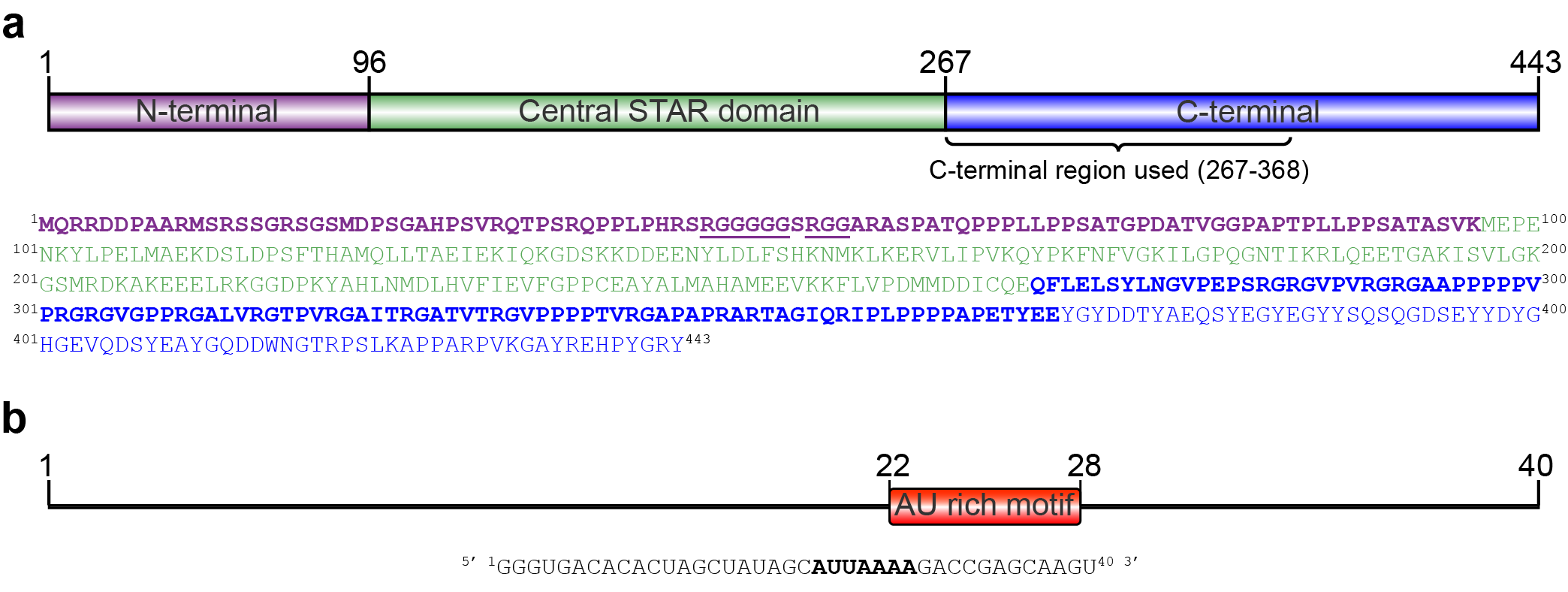
SI-12 Sequence information for Sam68 and G8.5 RNA**

Fig. S15| Sequence information for Sam68 and G8.5 RNA. **a**, Sequence for Sam68 coloured by domain. Residues for the N- and C-terminal used in this study highlighted in bold^34^. Bold and underlined residues represent the RNA binding site–the RG rich region–on the N-terminus. **b**, Sequence for G8.5 RNA^35^. Bold letters represent the AU rich region involved in binding to the RG rich region on the N-terminal. Illustrations were made using IBS^33^.

SI-13 Independent trapping traces of G8.5 RNA


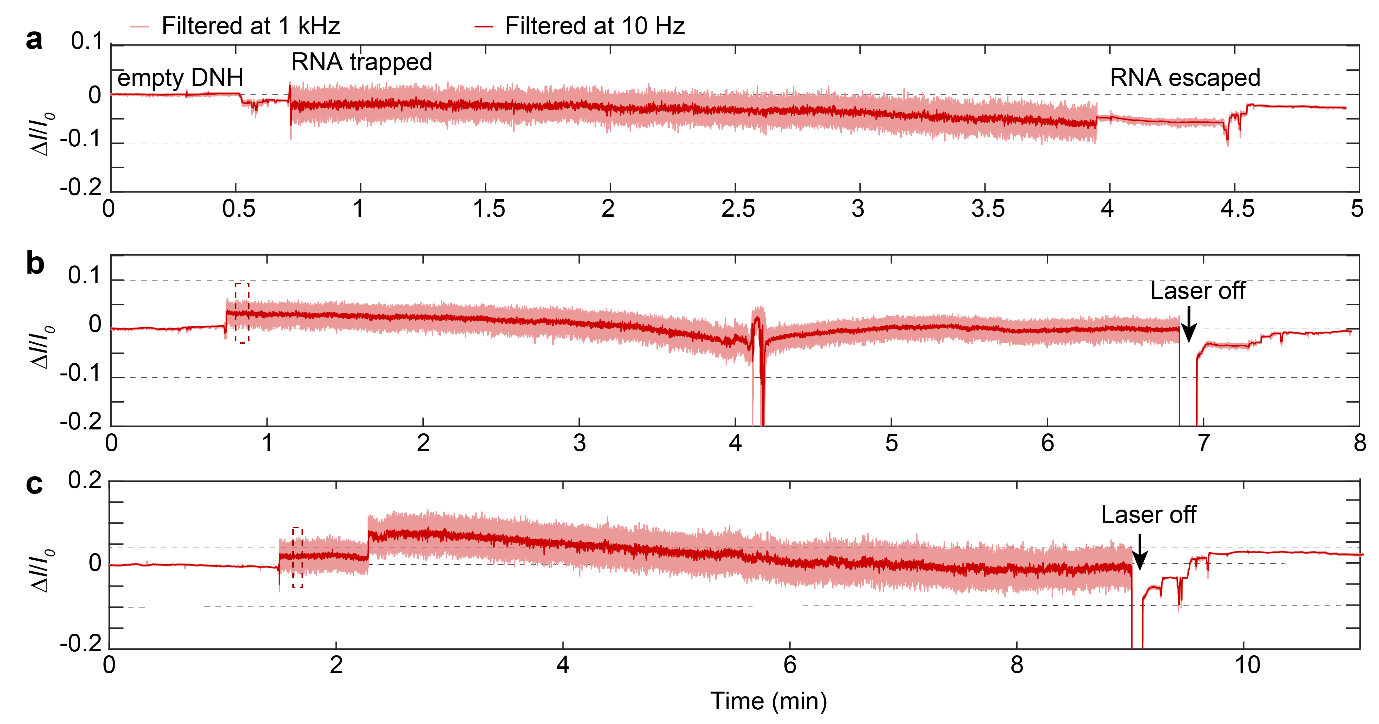


Fig. S16| G8.5 RNA trapping trajectories. **a,** Trapping trace where the RNA molecule escaped the trap. **b, c,** Trapping traces where the RNA molecule is released upon turning the laser off. The dashed boxes in **b** and **c** represent the section of the signal shown for RNA only in Figures 4c and S18c, respectively. Data shown were filtered at 1 kHz (pale red) and 10 Hz (dark red).

SI-14 Additional trapping traces of G8.5 RNA binding to the Sam68 N-terminal region

Two additional trapping experiments probing the binding of the Sam68 N-terminal region with G8.5 RNA showed a significant decrease in the dynamics of the Sam68 N-terminal region from RNA binding. For each experiment, we first trapped the Sam68 N-terminal region before infusing either 10 μM (Fig. S17) or 1 μM (Fig. S18). Figures S17a and S18a illustrate the full trapping trace for each individual experiment. For the trace presented in S17a, system instability warranted multiple realignment steps, illustrated by black arrows. Data were only analysed after the system stabilised following a realignment step, to minimise its impact on our data.

Figures S17b and S18b depict events where a signal shift occurs whilst the Sam68 N-terminal region is trapped and buffer containing G8.5 RNA is being infused. Each event was assigned two levels, with the lower level representing the trace when the Sam68 N-terminal region was trapped with no RNA, and an upper level assigned to formation of the Sam68-RNA complex. Notably, several segments did not fully match the signal change assigned to either the Sam68 N-terminal region or the Sam68-RNA complex being trapped (S17b, events 1 and 2, and S18b, events 3 and 4). These segments which lie between the assigned levels are attributed to be when both the Sam68 N-terminal region and G8.5 RNA are present within the trap, but have not fully associated with each other.

Figures S17c and S18c compares 5-second trace segments of the Sam68 N-terminal region without RNA bound to the signal when the Sam68-RNA complex is trapped. These segments are taken from the dashed boxes shown in Figures S17a and S18a for each dataset, respectively. S18c also includes a 5-second trace segment of G8.5 RNA alone, taken from the dashed box in Figure S16c. The traces in Figures S17c and S18c show the much higher level of dynamics exhibited by the Sam68 N-terminal region compared to the Sam68-RNA complex, supporting our conclusion that RNA binding induces stability and order to the Sam68 N-terminal region. Additionally, the G8.5 RNA trace in S18c also produces a signal with a similar level of fluctuation as the Sam68 N-terminal region, indicating it is a highly dynamic molecule, which is expected for RNA^36^. Figures S17d and S18d compare the PSDs of 20-second segments of data that S17c and S18c were derived from. S17d and S18d demonstrate a similar trend, where the Sam68-RNA demonstrates reduced signal fluctuations at frequencies below ~20 kHz (>50 μs), corresponding to increased order and stability induced by G8.5 RNA binding the Sam68 N-terminal region.

Notably, we also observed different binding kinetics in the trapping experiment shown in Figure S18. Binding of the Sam68 N-terminal region with G8.5 RNA still showed the same trend seen in our other experiments, where upon binding, the protein became more stable and ordered, and had several events where the RNA bound to the Sam68 N-terminal region before quickly dissociating and exiting the trap. However, for this trap, instead of the G8.5 RNA exiting the trap upon unbinding, the RNA molecule remained in the trap, allowing for continuous interactions between the Sam68 N-terminal region and G8.5 RNA (Figs. S18a and S18b).

**
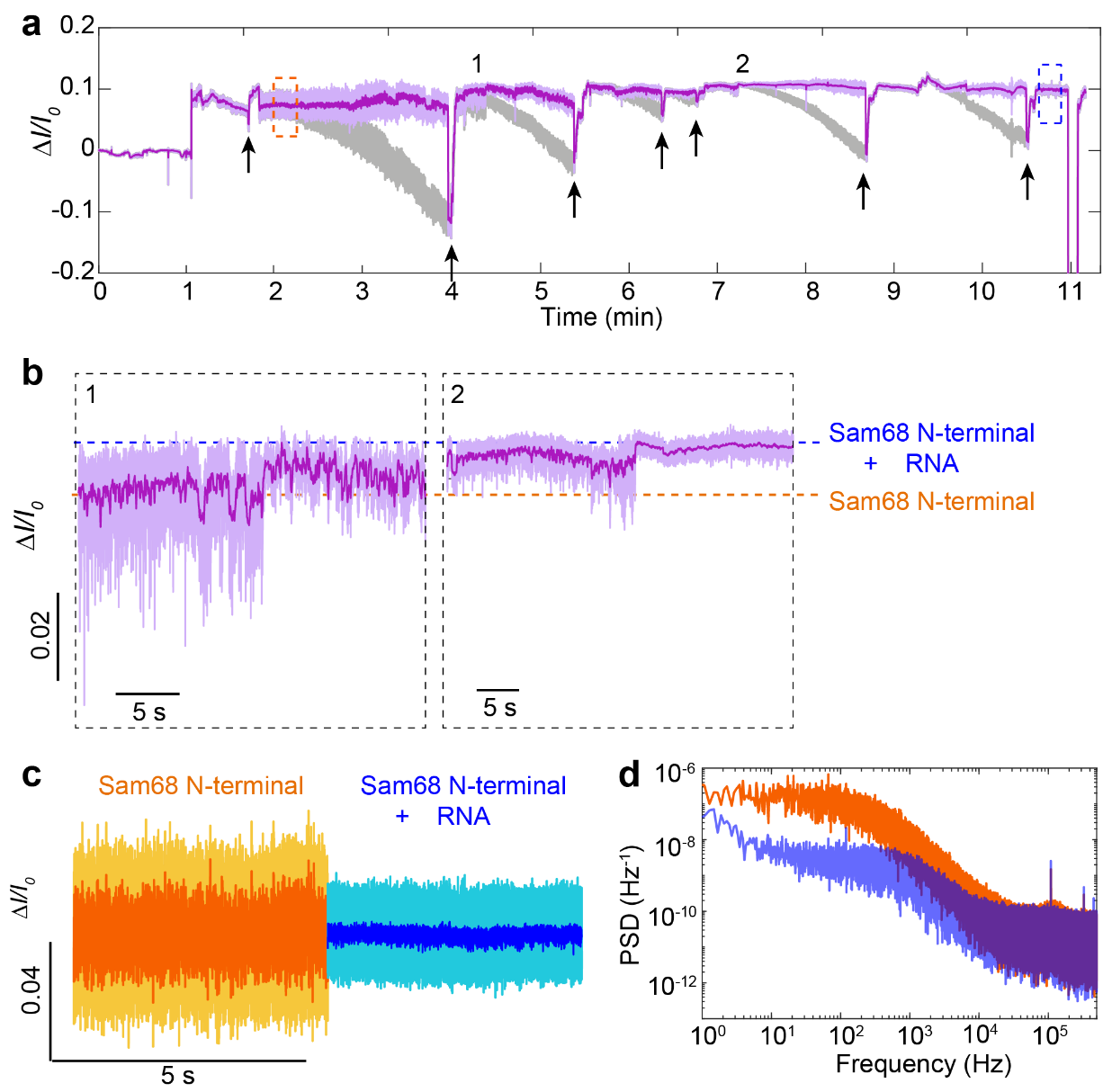
**

Fig. S17| Additional trace comparison of the Sam68 N-terminal region binding to G8.5 RNA. **a,** Detrended transmission trace of the Sam68 N-terminal region binding to G8.5 RNA. Raw trace shown in grey. Arrows indicate system re-alignment. **b,** Zoomed segments taken from **a**. Inset 1 shows the time when the G8.5 RNA enters the trap, whilst inset 2 shows when the G8.5 RNA fully binds to the Sam68 N-terminal region. Data shown were digitally filtered at 1 kHz (light purple) and 10 Hz (dark purple). **c,** Five-second trace comparison of when only the Sam68 N-terminal region was present in the trap (orange) versus when G8.5 RNA has bound to the protein (blue). Raw data shown at 1 MHz (light orange and light blue), and digitally filtered at 1 kHz (dark orange and dark blue). **d,** PSD plots for the trace segments in the dashed boxes shown in **a** for the Sam68 N-terminal region (orange, from orange dashed box) and when the Sam68 N-terminal region is bound to G8.5 RNA (blue, from blue dashed box).


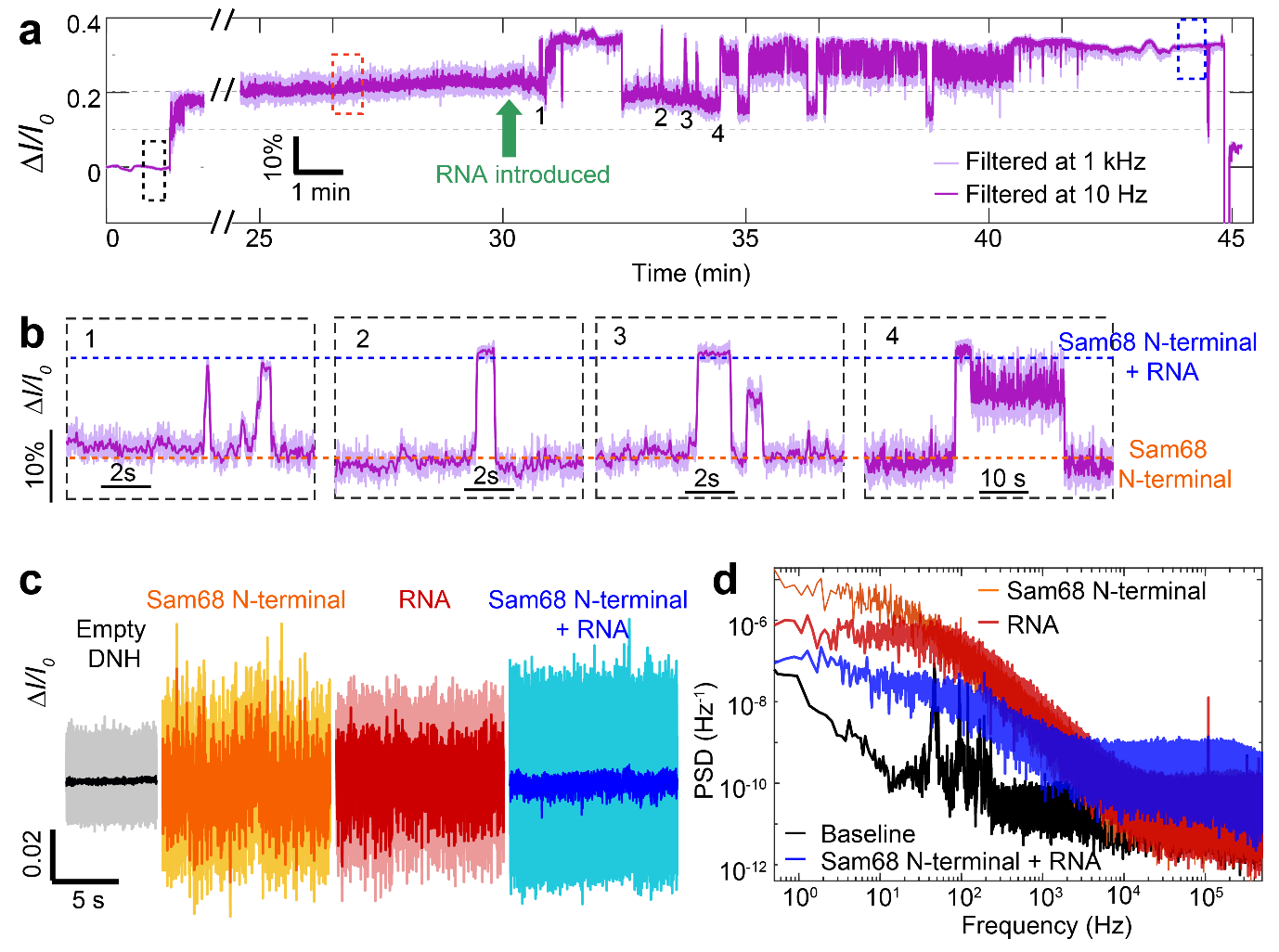


Fig. S18| Binding of G8.5 RNA to the Sam68 N-terminal region. **a**, Trapping trace of the Sam68 N-terminal region followed by binding/unbinding of RNA. Data was filtered to 1 kHz (light purple) and 10 Hz (dark purple). **b**, Four binding/unbinding events depicting the reduction in signal fluctuation of the Sam68 N-terminal region from before RNA binding (orange) and after forming the Sam68-RNA complex after G8.5 RNA has bound (blue). **c**, Zoomed segments from panel **a** depicting the Sam68 N-terminal region before RNA binding (orange, from orange dashed box) and the formed Sam68-RNA complex after RNA binding (blue, from blue dashed box). The RNA trace was acquired separately, with the full trace shown in Fig. S16c. Traces are shown in raw data (1 MHz sampling rate, light colours) and filtered data (1 kHz, dark colours). **d**, PSD of normalised traces for the baseline (black), the Sam68 N-terminal region before RNA binding (orange), G8.5 RNA (red) and the Sam68-RNA complex formed after RNA binding (blue) for comparison. Full trapping trace for **a** before detrending is provided in Fig S19.

**SI-15** Trapping trajectory of the Sam68 N-terminal before and after detrend


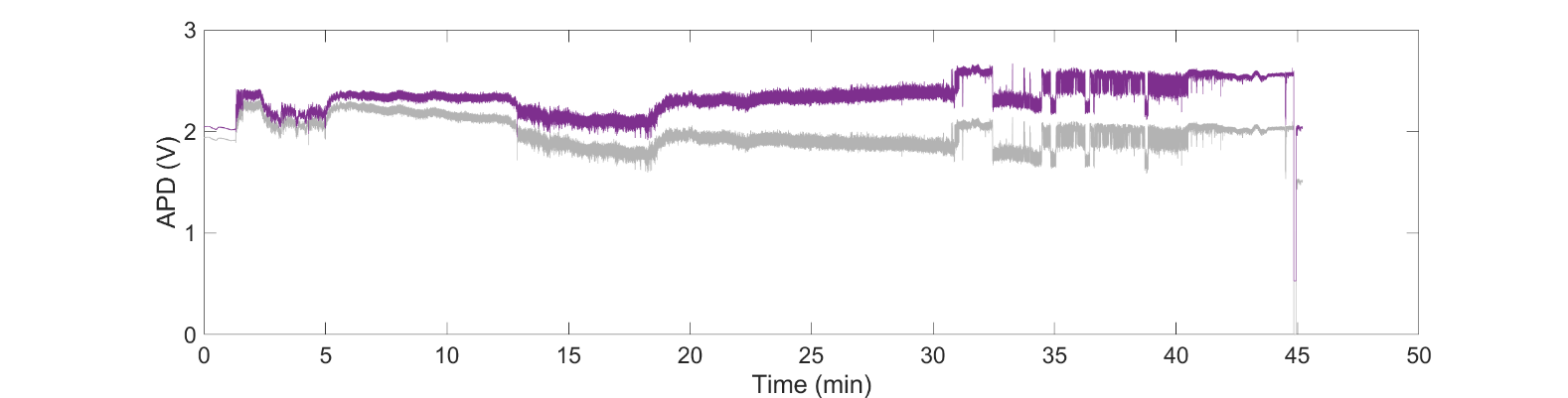


Fig. S19| Sam68 N-terminal region trapping trajectory with and without detrend. Raw (grey) and detrended (purple) transmission trace of the Sam68 N-terminal shown in Fig. S18a. Data shown were filtered at 1 kHz.

**References**

1. Ying, C. *et al.* Formation of Single Nanopores with Diameters of 20–50 nm in Silicon Nitride Membranes Using Laser-Assisted Controlled Breakdown. *ACS Nano* **12**, 11458–11470 (2018).

2. Quinten Michael. Beyond Mie’s Theory I – Nonspherical Particles. in *Optical Properties of Nanoparticle Systems* 255 (John Wiley & Sons, Ltd, 2011). doi:10.1002/9783527633135.ch9.

3. Pang, Y. & Gordon, R. Optical Trapping of a Single Protein. *Nano Lett.* **12**, 402–406 (2012).

4. Ying, C. *et al.* Watching Single Unmodified Enzymes at Work. Preprint at https://doi.org/10.48550/arXiv.2107.06407 (2021).

5. Peters, M. *et al.* Energy landscape of conformational changes for a single unmodified protein. *npj Biosensing* **1**, 1–10 (2024).

6. Born, M. & Wolf, E. *Principles of Optics: Electromagnetic Theory of Propagation, Interference and Diffraction of Light*. (Cambridge University Press, Cambridge, 1999).

7. Li, L., Li, C., Zhang, Z. & Alexov, E. On the Dielectric “Constant” of Proteins: Smooth Dielectric Function for Macromolecular Modeling and Its Implementation in DelPhi. *J. Chem. Theory Comput.* **9**, 2126–2136 (2013).

8. Sarimov, R. M., Matveyeva, T. A. & Binhi, V. N. Laser interferometry of the hydrolytic changes in protein solutions: the refractive index and hydration shells. *J Biol Phys* **44**, 345–360 (2018).

9. Booth, L. S. *et al.* Modelling of the dynamic polarizability of macromolecules for single-molecule optical biosensing. *Sci Rep* **12**, 1995 (2022).

10. Schuler, B. & Hofmann, H. Single-molecule spectroscopy of protein folding dynamics—expanding scope and timescales. *Current Opinion in Structural Biology* **23**, 36–47 (2013).

11. Mukrasch, M. D. *et al.* Structural Polymorphism of 441-Residue Tau at Single Residue Resolution. *PLOS Biology* **7**, e1000034 (2009).

12. Mylonas, E. *et al.* Domain Conformation of Tau Protein Studied by Solution Small-Angle X-ray Scattering. *Biochemistry* **47**, 10345–10353 (2008).

13. Toral-Rios, D., Pichardo-Rojas, P. S., Alonso-Vanegas, M. & Campos-Peña, V. GSK3β and Tau Protein in Alzheimer’s Disease and Epilepsy. *Front. Cell. Neurosci.* **14**, (2020).

14. Alquezar, C., Arya, S. & Kao, A. W. Tau Post-translational Modifications: Dynamic Transformers of Tau Function, Degradation, and Aggregation. *Front. Neurol.* **11**, (2021).

15. Basheer, N. *et al.* Does modulation of tau hyperphosphorylation represent a reasonable therapeutic strategy for Alzheimer’s disease? From preclinical studies to the clinical trials. *Mol Psychiatry* **28**, 2197–2214 (2023).

16. Beurel, E., Grieco, S. F. & Jope, R. S. Glycogen synthase kinase-3 (GSK3): Regulation, actions, and diseases. *Pharmacology & Therapeutics* **148**, 114–131 (2015).

17. Chatterjee, S., Sang, T.-K., Lawless, G. M. & Jackson, G. R. Dissociation of tau toxicity and phosphorylation: role of GSK-3β, MARK and Cdk5 in a Drosophila model. *Human Molecular Genetics* **18**, 164–177 (2009).

18. Darling, A. L. & Uversky, V. N. Intrinsic Disorder and Posttranslational Modifications: The Darker Side of the Biological Dark Matter. *Front. Genet.* **9**, (2018).

19. Marsh, J. A. & Forman-Kay, J. D. Sequence Determinants of Compaction in Intrinsically Disordered Proteins. *Biophysical Journal* **98**, 2383–2390 (2010).

20. Jin, F. & Gräter, F. How multisite phosphorylation impacts the conformations of intrinsically disordered proteins. *PLOS Computational Biology* **17**, e1008939 (2021).

21. Lasorsa, A. *et al.* Magnetic resonance investigation of conformational responses of tau protein to specific phosphorylation. *Biophysical Chemistry* **305**, 107155 (2024).

22. Jeganathan, S. *et al.* Proline-directed Pseudo-phosphorylation at AT8 and PHF1 Epitopes Induces a Compaction of the Paperclip Folding of Tau and Generates a Pathological (MC-1) Conformation *. *Journal of Biological Chemistry* **283**, 32066–32076 (2008).

23. Pearlman, S. M., Serber, Z. & Ferrell, J. E. A Mechanism for the Evolution of Phosphorylation Sites. *Cell* **147**, 934–946 (2011).

24. Chatterjee, S., Sang, T.-K., Lawless, G. M. & Jackson, G. R. Dissociation of tau toxicity and phosphorylation: role of GSK-3β, MARK and Cdk5 in a Drosophila model. *Human Molecular Genetics* **18**, 164–177 (2009).

25. Chakraborty, P. *et al.* GSK3β phosphorylation catalyzes the aggregation of tau into Alzheimer’s disease-like filaments. *Proceedings of the National Academy of Sciences* **121**, e2414176121 (2024).

26. El Hajjar, L. *et al.* Effect of PHF-1 hyperphosphorylation on the seeding activity of C-terminal Tau fragments. *Sci Rep* **15**, 9975 (2025).

27. Lin, Y.-T. *et al.* The binding and phosphorylation of Thr231 is critical for Tau’s hyperphosphorylation and functional regulation by glycogen synthase kinase 3β. *Journal of Neurochemistry* **103**, 802–813 (2007).

28. Structural Polymorphism of 441-Residue Tau at Single Residue Resolution | PLOS Biology. https://journals.plos.org/plosbiology/article?id=10.1371/journal.pbio.1000034.

29. Adzhubei, A. A., Sternberg, M. J. E. & Makarov, A. A. Polyproline-II Helix in Proteins: Structure and Function. *Journal of Molecular Biology* **425**, 2100–2132 (2013).

30. Chebrek, R., Leonard, S., de Brevern, A. G. & Gelly, J.-C. PolyprOnline: polyproline helix II and secondary structure assignment database. *Database* **2014**, bau102 (2014).

31. Zheng, M., Li, Z. & Huang, X. Ethylene Glycol Monolayer Protected Nanoparticles:  Synthesis, Characterization, and Interactions with Biological Molecules. *Langmuir* **20**, 4226–4235 (2004).

32. Local Structure and Dynamics of Hydration Water in Intrinsically Disordered Proteins | The Journal of Physical Chemistry B. https://pubs.acs.org/doi/10.1021/jp511961c.

33. IBS 2.0: an upgraded illustrator for the visualization of biological sequences | Nucleic Acids Research | Oxford Academic. https://academic.oup.com/nar/article/50/W1/W420/6586865.

34. Malki, I. *et al.* Cdk1-mediated threonine phosphorylation of Sam68 modulates its RNA binding, alternative splicing activity and cellular functions. *Nucleic Acids Research* **50**, 13045–13062 (2022).

35. Lin, Q., Taylor, S. J. & Shalloway, D. Specificity and Determinants of Sam68 RNA Binding: IMPLICATIONS FOR THE BIOLOGICAL FUNCTION OF K HOMOLOGY DOMAINS *. *Journal of Biological Chemistry* **272**, 27274–27280 (1997).

36. Spitale, R. C. & Incarnato, D. Probing the dynamic RNA structurome and its functions. *Nat Rev Genet* **24**, 178–196 (2023).
